# Supplementary material for: Spectral phase transitions in optical parametric oscillators
Source: Nat Commun. 2021 Feb 5;12:835. doi: 10.1038/s41467-021-21048-z (PMC7864919; doi:10.1038/s41467-021-21048-z)
Supplement: Supplementary file 1 — Supplementary Information [file 41467_2021_21048_MOESM1_ESM.pdf]

Supplemental Information: Spectral Phase Transitions in Optical Parametric Oscillators

Arkadev Roy[1], Saman Jahani[1], Carsten Langrock[2], Martin Fejer[2], and Alireza Marandi[1*]

[1] Department of Electrical Engineering, California Institute of Technology, Pasadena, California 91125, USA

[2]Edward L. Ginzton Laboratory, Stanford University, Stanford, California, 94305, USA

December 30, 2020

1 Parameters used in Simulation:

Here, we tabulate the system parameters that is used in the simulation.

Table 1: Parameters for the PPLN waveguide DROPO and pump pulse.

Symbol	Meaning	Value	Unit
$\lambda^{(a)}$	Signal Wavelength	1550	nm
$\lambda^{(b)}$	Pump Wavelength	775	nm
L	Waveguide Length	40	mm
$\alpha^{(a)}$, $\alpha^{(b)}$	Waveguide Loss	0.00691	mm^{-1}
u	Walk off/ GVM	0.329	ps/mm
T_p	Pump Pulse Width	12	ps
$\beta_2^{(a)}$	Signal GVD	1.12 x 10^{-4}	ps^2/mm
$\beta_3^{(a)}$	Signal TOD	3.09 x 10^{-7}	ps^3/mm
$\beta_2^{(b)}$	Pump GVD	4.06 x 10^{-4}	ps^2/mm
$\beta_3^{(b)}$	Pump TOD	2.51 x 10^{-7}	ps^{3}mm
ϵ[1]	Effective nonlinearity	5.16 x 10^{-5}	ps$^{\frac{1}{2}}/mm$
G_0[2]	Power gain at threshold	7.9	
b_0[3]	CW threshold amplitude	5.29 X 10^2	ps$^{-\frac{1}{2}}$
$N_{b,0}$[4]	Threshold photons	3.36 x 10^6	

*marandi@caltech.edu

[1] $\epsilon = \sqrt{2\hbar\omega\eta}$. $\eta = 100\% W^{-1}cm^{-2}$ is the normalized SHG conversion efficiency.

[2] $(1 - G_0^{-1})$ is the lumped round-trip loss. Here we assume a 9-dB per round-trip loss.

[3] $b_0 = \frac{\alpha^{(b)}}{4\epsilon}(e^{\alpha^{(b)}\frac{L}{2}} - 1)^{-1} ln(G_0 e^{\alpha^{(a)}L})$.

[4] $N_{b,0} = T_p b_0^2$.

2 Additional Experimental Details:

To obtain the optical spectrum in scanning mode we used a tunable filter (1 nm bandwidth) in order to record a vertical slice of Fig2.c. The photo-detected unfiltered signal is triggered using the functionality of Sequence B Trigger (B Trigger after A events) of the oscilloscope (Tektronix MD04104-6). Here, the scanning ramp signal comprises the event A, and the unfiltered OPO output plays the role of event B.

The round-trip loss of the cavity amounts to approximately 9 dB. All fibers and devices existing in the optical path are single mode, polarization maintaining and connectors are angle polished. A tunable band-pass filter (Agiltron FOTF) is used as a monochromator. A combination of a Fast Detector (Menlo Systems FPD-510) and RF-Spectrum Analyser is used to measure the radio-frequency spectrum after beating the OPO output with that of the fully stabilized frequency comb (Menlo Systems FC1500-250-WG). In our configuration we have 4 pulses circulating per round-trip.

The threshold in our setup is observed to be 680 μW of average power. The measurement is taken at a pump average power of 820 μW . This implies $p \approx 1.1$. The pump is centered around 775 nm (approximately 12 ps long pulses, 250 MHz repetition rate). The pigtail of the PPLN waveguide measures 2 m in length and is made up of single mode, polarization maintaining (PM) fiber. Additionally, we have another PM fiber of length 15 cm. The combination of the pigtail, non poled portion of the waveguide, fiber section of the phase shifter and the additional fiber contributes to ϕ_2.

Our methodology of capturing the phase transition in scanning mode (quasi-static) enable us to observe the critical point (CP), an aspect which lacked unambiguous demonstration as far as phase transition under the purview of laser physics is concerned. Our ability to operate in the locked mode and bias the system around the critical point opens up new possibilities to realize outstanding sensing arrangement based on phase transition.

We measure the radio-frequency spectrum/beat-note by beating the OPO output with that of the broadband fully stabilized frequency comb (Menlo Comb). On account of the pulsed nature, we had to adjust the delay line in order to temporally overlap the OPO output and the fully stabilized frequency comb output.

This enhanced sensitivity feature of spectral phase transition holds promise for the development of next generation transition edge sensors. In this endeavour, an essential requirement is to operate the OPO in locked mode with the aid of an auxiliary laser. We present here results of the spectral phase transition (Fig S.2), where spectrum at different detuning positions are measured, while keeping the OPO locked at these discrete detuning values. Portion of the schematic (Fig S.1) encompassed by dotted lines are deployed for locking mode operation. Tunable continuous wave laser (Orbits Lightwave, 1550 nm) output is injected into the cavity in a direction opposite to the circulation of the signal. PID control is performed after extracting the error signal using the Pound-Drever-Hall technique. The optical spectrum of the OPO locked at different cavity detuning is displayed in Fig S.2. The auxiliary CW laser used to lock the cavity appears as a notch in the spectrum at 1550 nm.

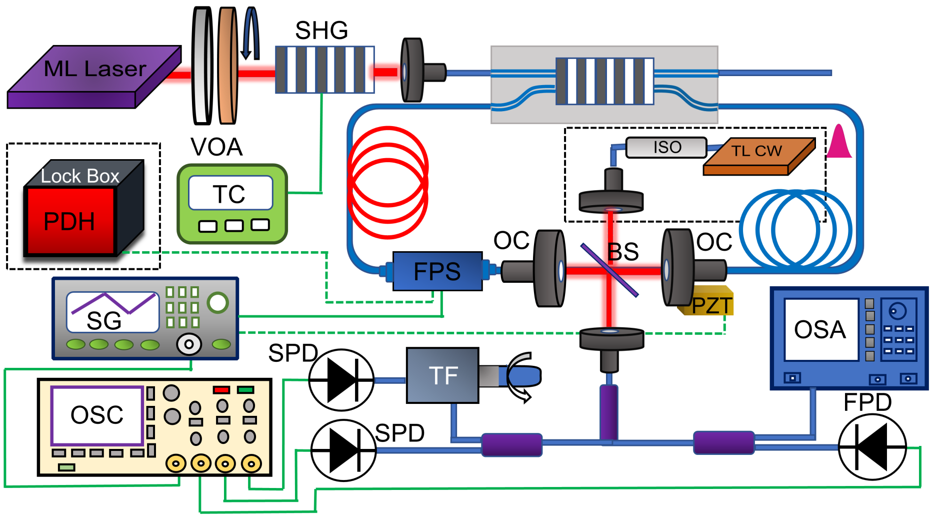

Figure S.1: Detailed experimental schematic showing all optical pathways, electrical connections, and locking arrangement. Components included inside the dashed boundary are deployed for locking arrangement. OSC: Oscilloscope, SG: Signal Generator, TF: Tunable Band-pass filter, OSA: Optical Spectrum Analyser, ISO: Isolator, OC: Output Coupler, FPS: Fiber phase shifter(General Photonics-FPS-001), ML Laser: Mode Locked Laser (Menlo Systems FC1500-250-WG, 250 MHz repetition rate, 1550 nm), SHG: Second Harmonic Generation (Covesion MSHG1550-1.0-40), SPD: Slow Photo Detector, FPD: Fast Photo Detector, TC: Temperature Controller(Covesion OC2 Temperature Controller), BS: Beam Splitter, VOA: Variable Optical Attenuator, PZT: Piezo Transducer, PDH: Pound Drever Hall Lock Box, TL CW: Tunable Continous Wave Laser.

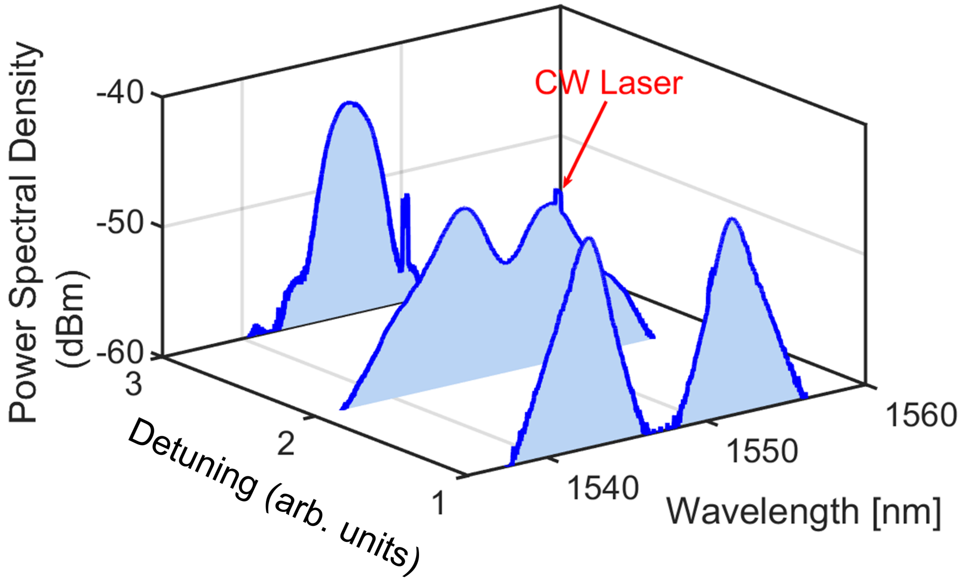

Figure S.2: Spectral phase transition from non-degenerate to degenerate regime captured experimentally in locked configuration.

3 Derivation of high Finesse limit OPO mean-field evolution equation:

Here, we show the derivation of the mean-field equation that appears in Eq 1 of the main text.

In the high finesse limit we consider the doubly resonant OPO to be governed by parametric nonlinear interaction, group velocity dispersion (cavity averaged) induced chirp, round-trip loss, and detuning of the cavity. The quadratic nonlinear interaction considered is given by Eq(S.1). Eq(S.1b) can be integrated by the method of characteristics to yield Eq(S.2a). This when substituted in Eq(1a) gives Eq(S.2b). The resultant nonlinear interaction is summarized in Eq(S.3a), while the effect of GVD is included in Eq(S.3b). Combining all the relevant cavity effect we get the evolution of the signal field on a round-trip basis as given in Eq(S.4a). Under the assumptions of the high finesse cavity, we convert the difference equation to a partial differential equation (S.4b). We define several normalization factors (Eq(S.5)) and express the signal evolution in the mean-field approximation by Eq(S.6).

$$\frac{\partial a}{\partial z} = \epsilon a^* b \tag{S.1a}$$

$$\frac{\partial b}{\partial z} = -u\frac{\partial b}{\partial t} - \frac{\epsilon}{2}a^2 \tag{S.1b}$$

$$b(z,t) = b_0(t-uz) - \frac{\epsilon}{2u}\int_{t-uz}^{t} a(z - \frac{t-t'}{u}, t')^2 dt' \tag{S.2a}$$

$$\frac{\partial a(z,t)}{\partial z} = \epsilon b_0(t-uz)a(z,t)^* - \frac{\epsilon^2}{2u}a(z,t)^* \int_{t-uz}^{t} a(z - \frac{t-t'}{u}, t')^2 dt' \tag{S.2b}$$

$$a_{\rm out}(t) = a_{\rm in}(t) + \left[\frac{\epsilon}{u}\int_0^{Lu} b_0(t-\tau)d\tau\right] a^*_{\rm in}(t) - \left[\frac{\epsilon^2}{2u^2}\int_0^{Lu}(Lu-\tau)a_{\rm in}(t-\tau)^2 d\tau\right] a^*_{\rm in}(t) \tag{S.3a}$$

$$\frac{da}{dt} = \left[-i\frac{\beta_2}{2!}\frac{\partial^2}{\partial t^2} + \frac{\beta_3}{3!}\frac{\partial^3}{\partial t^3}\right] a(t) \tag{S.3b}$$

$$\begin{aligned} a_{n+1}(t) - a_n(t) &= (-\alpha + i\Delta\phi)a_n(t) + \left[\frac{\epsilon}{u}\int_0^{Lu} b_0(t-\tau)d\tau - \frac{\epsilon^2}{2u^2}\int_0^{Lu}(Lu-\tau)a_n(t-\tau)^2 d\tau\right] a^*_n(t) \\ &\quad + \left[-i\frac{\beta_2}{2!}\frac{\partial^2}{\partial t^2} + \frac{\beta_3}{3!}\frac{\partial^3}{\partial t^3}\right] a_n(t) \end{aligned} \tag{S.4a}$$

$$\begin{aligned} T_R\frac{\partial a(\xi,t)}{\partial \xi} &= (-\alpha + i\Delta\phi)a(\xi,t) + \left[\frac{\epsilon}{u}\int_0^{Lu} b_0(t-\tau)d\tau - \frac{\epsilon^2}{2u^2}\int_0^{Lu}(Lu-\tau)a(t-\tau)^2 d\tau\right] a^*(\xi,t) \\ &\quad + \left[-i\frac{\beta_2}{2!}\frac{\partial^2}{\partial t^2} + \frac{\beta_3}{3!}\frac{\partial^3}{\partial t^3}\right] a(\xi,t) \end{aligned} \tag{S.4b}$$

$$\xi' = \frac{\xi}{T_R}, \quad g = \frac{\epsilon}{u}\int_0^{Lu} b_0(t-\tau)d\tau \tag{S.5}$$

$$\frac{\partial a}{\partial \xi} = (-\alpha + i\Delta\phi)a + ga^* - \left[\frac{\epsilon^2}{2u^2}\int_0^{Lu}(Lu-\tau)a(t-\tau)^2 d\tau\right]a^* - i\frac{\beta_2}{2}\frac{\partial^2 a}{\partial t^2} \tag{S.6}$$

4 CW gain limit of OPO (without mean-field approximation):

While the high Q limit (where mean-field approximation is accurate), Eq S.6 can predict the occurrence of the spectral phase transition, it also occurs in the low Q limit (where mean field approximation is not valid and lumped interaction is to be considered) as well. In fact, our experimental realization deals with low Finesse cavity. In such scenarios where the mean-field equation is no more valid, we present a model that captures the spectral phase transition assuming that the pump is CW. Note that, the analysis for the pulsed pumping case is not trivial, and one has to resort to full numerical simulation.

Reference [1] suggest that the CW gain approximation can predict the frequency of oscillation with a good agreement. However, one has to include the gain clipping component as well to obtain the signal/idler pulse shapes, power spectrum in case of synchronously pumped OPO. Thus for studying spectral phase transition, the CW gain limit is a reliable approach. In any case, one can always perform full spatio-temporal simulation to judge if the approximation holds good or not.

We assume signal (a_s) to be centered at frequency $\omega_0 + \delta\omega$, and idler (a_i) at $\omega_0 - \delta\omega$, when the pump frequency is $2\omega_0$. We define: $a_+ = \frac{(a_s + a_i^*)}{2}$, and $a_- = \frac{(a_s - a_i^*)}{2}$. In the near-threshold limit, we neglect the effect of gain saturation. From Eq(1a), of the main text, we obtain:

$$\begin{bmatrix} \dot{a_+} \\ \dot{a_-} \end{bmatrix} = \begin{bmatrix} -\frac{\alpha^{(a)}}{2} + \epsilon b & i\frac{\beta_2^{(a)}(\delta\omega)^2}{2} \\ i\frac{\beta_2^{(a)}(\delta\omega)^2}{2} & -\frac{\alpha^{(a)}}{2} - \epsilon b \end{bmatrix} \begin{bmatrix} a_+ \\ a_- \end{bmatrix} \tag{S.7}$$

Eq(S.7) describes the interaction within the PPLN waveguide. After exiting the gain medium, the signal/idler fields encounter the cavity dispersion, out-coupling loss, and round-trip feedback. These interactions outside the gain medium is governed by Eq(2) of main text. This leads to: $a_s \rightarrow G_0^{-\frac{1}{2}} e^{i(\phi+\psi)} a_s$, $a_i \rightarrow G_0^{-\frac{1}{2}} e^{i(\phi-\psi)} a_i$, where $\phi = \Delta\phi + \frac{1}{2}\phi_2(\delta\omega)^2$ (symmetric phase shift) and $\psi = \frac{l\lambda^{(a)}}{2c}\delta\omega$ (asymmetric phase shift) .

$$\begin{bmatrix} a_+ \\ a_- \end{bmatrix}_{n+1} \rightarrow G_0^{-\frac{1}{2}} e^{-\frac{\alpha^{(a)}L}{2}} e^{i\psi} \begin{bmatrix} \cos(\phi) & i\sin(\phi) \\ i\sin(\phi) & \cos(\phi) \end{bmatrix} exp\left(\begin{bmatrix} \epsilon b & i\frac{\beta_2^{(a)}(\delta\omega)^2}{2} \\ i\frac{\beta_2^{(a)}(\delta\omega)^2}{2} & -\epsilon b \end{bmatrix} L \right) \begin{bmatrix} a_+ \\ a_- \end{bmatrix}_n \tag{S.8}$$

The round-trip evolution of $a_\pm$ is dictated by Eq(S.8). This equation has two eigenvalues: $\lambda_\pm$. The larger of them (λ_+) denotes the round-trip gain. The frequency of oscillation ($\delta\omega$) will be determined by $\lambda_{\max}$.

One can posit that, this essentially happens when the phase shift ($\Delta\phi$) is compensated by the total GVD (waveguide + cavity) contribution ($\beta_2 = \phi_2 + \beta_2^{(a)}L$) and is given by Eq(S.9).

$$\Delta\phi + \frac{\beta_2}{2}(\delta\omega)^2 = n\pi \tag{S.9}$$

The zeroth order resonance condition gives $\Delta\phi + \frac{\beta_2}{2}(\delta\omega)^2 = 0$. This means in the non-degenerate regime: $\delta\omega = \sqrt{-\frac{2\Delta\phi}{\beta_2}}$. When the perturbation is caused about the critical point ($\Delta\phi = 0$), we can write $\Delta\phi = \epsilon$, where ϵ is the small perturbation. This gives the square-root dependence of frequency splitting (similar to second-order exceptional point) for sensors based on second-order spectral phase transition, biased at critical point.

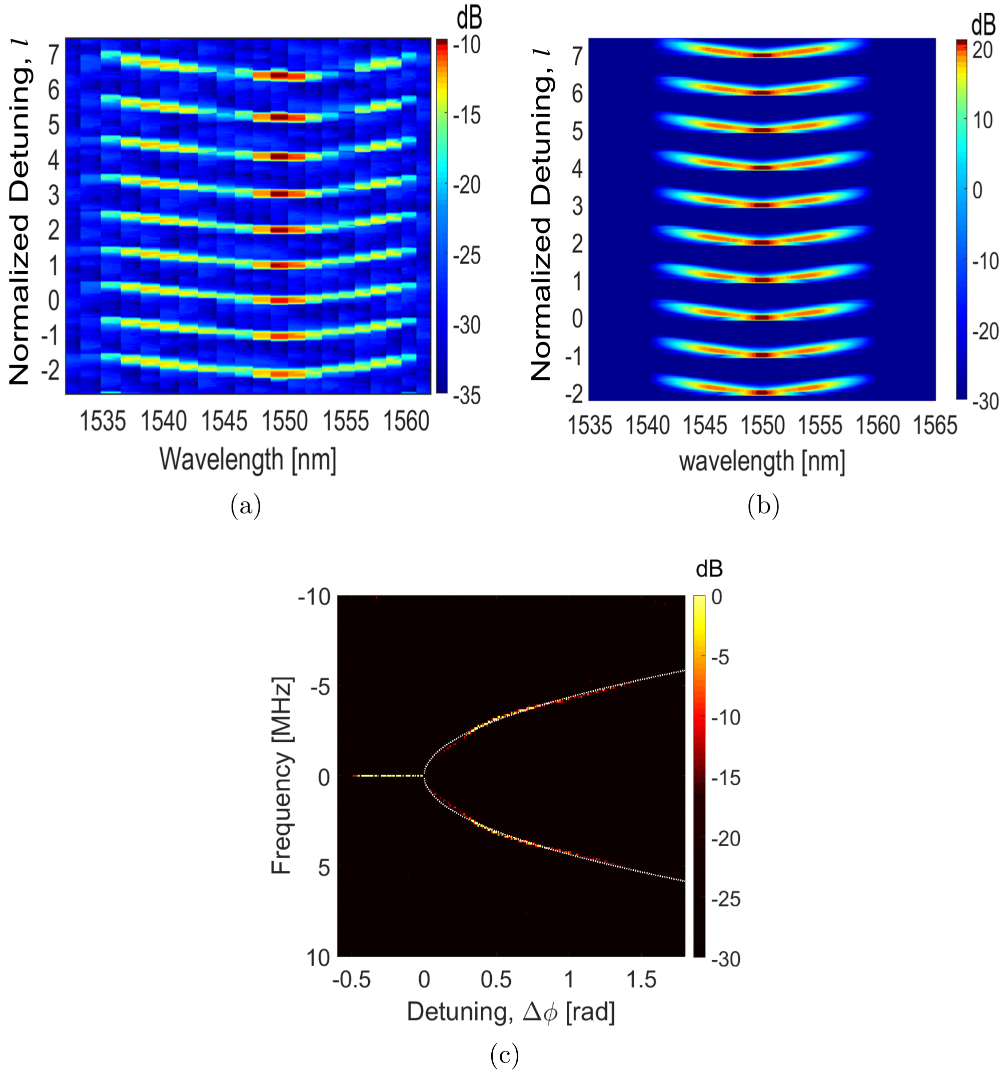

Figure S.3: Resonance Diagram for different orders. a) Experimental plot showing various orders of resonances centered around integer values of normalized detuning, l. b) The corresponding plot obtained through numerical simulation. c) Spontaneous emergence of beat-note around the critical point. Measured RF spectrum captured using a fast detector. The RF-spectral splitting closely follows a square-root behaviour around the critical point as evident by the dotted curve.

5 Effect of Group Velocity Dispersion:

The spectral splitting in the non-degenerate regime that accompanies the spectral phase transition can be engineered. Here, we study how the spectral splitting is dependant on the cavity dispersion.

From Eq(S.9) it is evident that the spectral splitting in the non-degenerate regime is a function of the second order (β_2) group velocity dispersion (GVD) for a fixed detuning. The sensitivity of sensors based on this spectral phase transition will be higher in cavities with lower β_2. At the same time we also find, that GVD influences the gain and thereby the order parameter in the non-degenerate phase. The change in slope of the order parameter for different values of GVD also highlights the variation of the sensitivity.

To investigate the effect of GVD on the spectral splitting, we assume that the cavity is homogeneous with finite β_2. Higher order dispersions are neglected. Fig S.4a suggests that the spectral splitting diverges as it approaches zero GVD. However, in practice higher order dispersions will contribute in the limit of near zero β_2. Also, the dispersion of nonlinearity needs to be accounted for distantly separated signal/idler peaks. In the near zero GVD limit the lumped assumption of $\beta_2^{(a)}$ and ϕ_2 contributing to net GVD β_2 breaks down, and Eq(S.9) cease to be valid. One has to resort to numerical simulation to study this scenario. For Fig(S.4b) and Fig(S.4c) we are operating away from near zero GVD, and the lumped approximation is valid. We thus change the net GVD by varying ϕ_2.

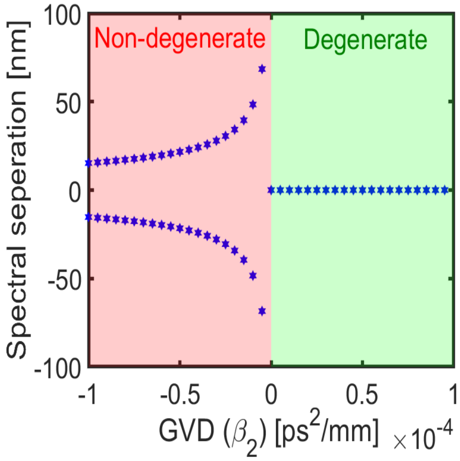

(a)

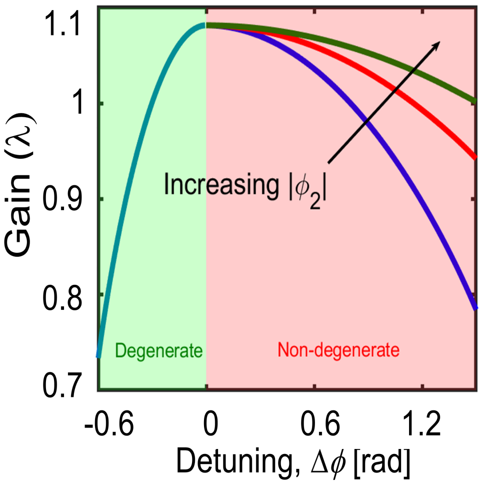

(b)

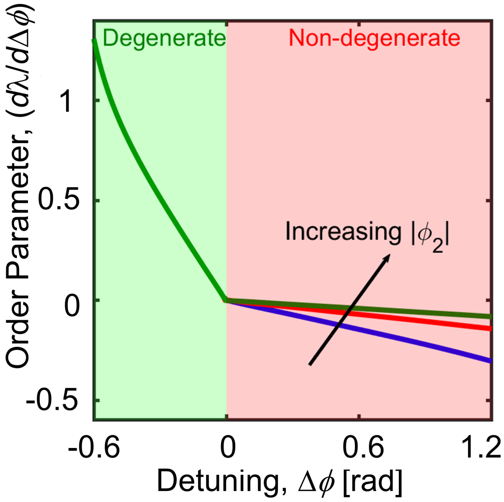

(c)

Figure S.4: Effect of GVD on Spectral phase transition. a) Spectral splitting as a function of cavity GVD. The detuning is kept fixed at $\Delta\phi = 0.3$. The dependence of gain (b) and order parameter (c) as the GVD is varied. These results are obtained from numerical simulation.

6 Description of Coupled OPO, CW Gain limit (without Mean-Field approximation):

Here, we consider an analytical model that captures the occurrence of first-order spectral phase transition in coupled OPO. This assumes the pump to be CW, and does not assume validity of mean-field approximation.

We consider two identical OPO's coupled with each other via conservative coupling.They can be

realized in a form as shown in Fig(S.5a). Here the clockwise(CW, A) and counter-clockwise (CCW, B) modes comprise the coupled OPO. Coupled OPO's realized in different cavities with non-identical parameters is also excepted to show similar qualitative behaviour.

We assume signal (A_s, B_s) to be centered at frequency $\omega_0 + \delta\omega$, and idler (A_i, B_i) at $\omega_0 - \delta\omega$, when the pump frequency is $2\omega_0$. We define: $A_+ = \frac{(A_s + A_i^*)}{2}$, $A_- = \frac{(A_s - A_i^*)}{2}$, $A_+ = \frac{(A_s + A_i^*)}{2}$, and $A_- = \frac{(A_s - A_i^*)}{2}$. Apart from the usual effects of parametric gain, out-coupling loss, cavity feedback, cavity dispersion that is encountered in a single OPO configuration, there exists the effect of conservative coupling (κ) which is given by Eq(S.10).The round-trip evolution of $A_\pm, B_\pm$ is dictated by Eq(S.11).We are interested in the eigenvalues of this evolution equation.

$$\begin{bmatrix} A_s \\ A_i \\ B_s \\ B_i \end{bmatrix} \rightarrow \begin{bmatrix} \sqrt{1-\kappa^2} & 0 & i\kappa & 0 \\ 0 & \sqrt{1-\kappa^2} & 0 & i\kappa \\ i\kappa & 0 & \sqrt{1-\kappa^2} & 0 \\ 0 & i\kappa & 0 & \sqrt{1-\kappa^2} \end{bmatrix} \begin{bmatrix} A_s \\ A_i \\ B_s \\ B_i \end{bmatrix} \tag{S.10}$$

$$\begin{aligned} \begin{bmatrix} A_+ \\ A_- \\ B_+ \\ B_- \end{bmatrix}_{n+1} \rightarrow G_0^{-\frac{1}{2}} e^{-\frac{\alpha^{(a)} L}{2}} e^{i\psi} & \begin{bmatrix} \sqrt{1-\kappa^2} & 0 & 0 & i\kappa \\ 0 & \sqrt{1-\kappa^2} & i\kappa & 0 \\ 0 & i\kappa & \sqrt{1-\kappa^2} & 0 \\ i\kappa & 0 & 0 & \sqrt{1-\kappa^2} \end{bmatrix} \\ & \begin{bmatrix} \cos(\phi) & i\sin(\phi) & 0 & 0 \\ i\sin(\phi) & \cos(\phi) & 0 & 0 \\ 0 & 0 & \cos(\phi) & i\sin(\phi) \\ 0 & 0 & i\sin(\phi) & \cos(\phi) \end{bmatrix} \\ exp & \left(\begin{bmatrix} \epsilon b & i\frac{\beta_2^{(a)}(\delta\omega)^2}{2} & 0 & 0 \\ i\frac{\beta_2^{(a)}(\delta\omega)^2}{2} & -\epsilon b & 0 & 0 \\ 0 & 0 & \epsilon b & i\frac{\beta_2^{(a)}(\delta\omega)^2}{2} \\ 0 & 0 & i\frac{\beta_2^{(a)}(\delta\omega)^2}{2} & -\epsilon b \end{bmatrix} L \right) \begin{bmatrix} A_+ \\ A_- \\ B_+ \\ B_- \end{bmatrix}_n \end{aligned} \tag{S.11}$$

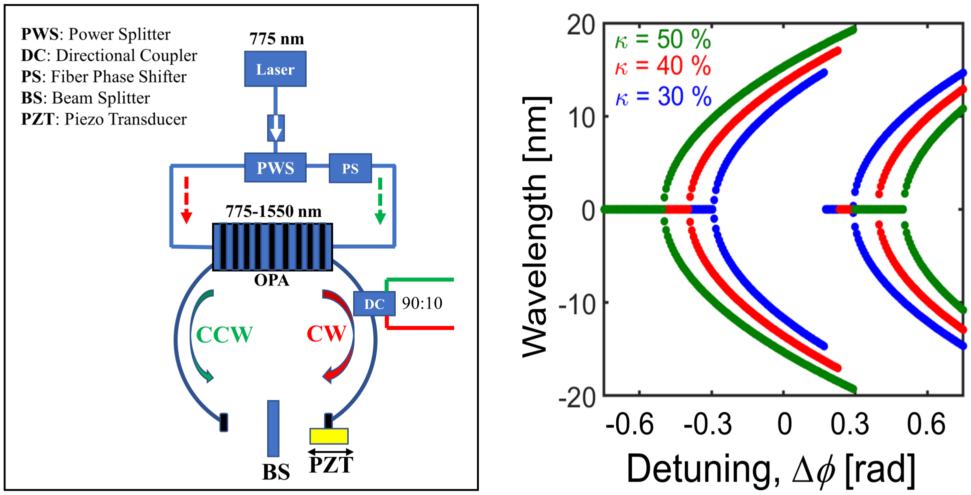

(a) (b)

Figure S.5: a) Schematic of a coupled OPO arrangement leveraging the clockwise(CW) and counter clockwise (CCW) degrees of freedom for a single resonator. b) Effect of different coupling strength is studied. Clearly, with increase in the coupling strength the two second order critical points move apart.

7 Description of Coupled OPO, Mean-Field approximation:

Here, we present the mean-field version of coupled OPO modelling that is capable of predicting the occurrence of first order spectral phase transition.

The CW driven, high finesse limit leading to the mean-field approximation in case of coupled OPO is unable to predict the occurrence of the first order phase transition. This is because in the mean-field limit it is assumed that the detuning is very small and only a truncated approximation of the detuning is included in the modelling equation. However, it is able to predict the existence of two second-order phase transition each centered around the split-mode resonance (mode-splitting/ avoided mode crossing accompanies dispersively coupled cavities). It is evident that first-order transition happens as a result of the interplay between the two second-order phase transitions.

Here, a and b denotes the signal amplitude of the individual OPO's comprising the coupled OPO. They are assumed to have identical detuning ($\Delta\phi$), cavity averaged GVD (β_2). Higher order dispersion terms are neglected. ξ and t represents the slow time and fast time respectively. The parametric gain term are expressed as p and q where they can be related as $q = pe^{i\theta}$ without loss of generality. The coupling term is designated as κ.

$$\frac{\partial a}{\partial \xi} = (-\alpha + i\Delta\phi)a + pa^* - \left[\frac{\epsilon^2}{2u^2}\int_0^{Lu}(Lu-\tau)a(t-\tau)^2 d\tau\right]a^* - i\frac{\beta_2}{2}\frac{\partial^2 a}{\partial t^2} + i\kappa b \tag{S.12a}$$

$$\frac{\partial b}{\partial \xi} = (-\alpha + i\Delta\phi)b + qb^* - \left[\frac{\epsilon^2}{2u^2}\int_0^{Lu}(Lu-\tau)b(t-\tau)^2 d\tau\right]b^* - i\frac{\beta_2}{2}\frac{\partial^2 b}{\partial t^2} + i\kappa a \tag{S.12b}$$

We assume a perturbation of the form as given in Eq(S.13a). We perform the linear stability analysis (Eq(S.13b)), where the gain saturation term has been neglected. We define $\Gamma = \Delta\phi + \frac{\beta_2}{2}(\delta\omega)^2$. The

most unstable eigenvalue that determines the CW oscillation threshold, and the frequency of oscillation is given by Eq(S.13c). Under the assumption that $|q| = |p|$, Eq(S.13c) reduces to Eq(S.13d).

$$a = a_+ e^{i\delta\omega t} + a_- e^{-i\delta\omega t}, \quad b = b_+ e^{i\delta\omega t} + b_- e^{-i\delta\omega t} \tag{S.13a}$$

$$\frac{d}{d\xi}\begin{bmatrix} a_+ \\ a_-^* \\ b_+ \\ b_-^* \end{bmatrix} = \begin{bmatrix} -\alpha + i(\Delta\phi + \frac{\beta_2}{2}(\delta\omega)^2) & p & i\kappa & 0 \\ p^* & -\alpha - i(\Delta\phi + \frac{\beta_2}{2}(\delta\omega)^2) & 0 & -i\kappa \\ i\kappa & 0 & -\alpha + i(\Delta\phi + \frac{\beta_2}{2}(\delta\omega)^2) & q \\ 0 & -i\kappa & q^* & -\alpha - i(\Delta\phi + \frac{\beta_2}{2}(\delta\omega)^2) \end{bmatrix} \begin{bmatrix} a_+ \\ a_-^* \\ b_+ \\ b_-^* \end{bmatrix} \tag{S.13b}$$

$$2(\lambda_{\max} + \alpha)^2 = |p|^2 + |q|^2 - 2\Gamma^2 - 2\kappa^2 + \sqrt{(|p|^2 - |q|^2)^2 - 4\kappa^2(|p|^2 + |q|^2) + 4\kappa^2(pq^* + p^*q) + 16\Gamma^2\kappa^2} \tag{S.13c}$$

$$(\lambda_{\max} + \alpha)^2 = |p|^2 - \Gamma^2 - \kappa^2 + 2\kappa\sqrt{\Gamma^2 - |p|^2 \sin^2(\frac{\theta}{2})} \tag{S.13d}$$

Clearly, we can obtain the presence of two second-order spectral phase transition points (assuming $\theta = 0$): one located at $\Delta\phi = \kappa$ and the other at $\Delta\phi = -\kappa$.

8 Swift- Hohenberg Equation/ Order Parameter Description:

Diverse nonlinear systems in laser physics although being described by distinct equations, sometimes exhibit identical nonlinear dynamics. All these systems, can be reduced to a normal form under certain assumptions, which is also known as the order parameter description or the Swift-Hohenberg equation (SHE)[2]. SHE provides a generalized framework to study pattern formation/ localized structures.

$$\frac{\partial A}{\partial t} = \underbrace{\mu A - qA^3}_{\substack{\text{super-critical} \\ \text{pitchfork bifurcation}}} \underbrace{- r(\nabla^2 + \Omega_0^2)^2 A}_{\text{SPT}}$$

We provide the derivation for the real SHE here. Here we assume a crude approximation of the gain saturation term and represent it by $q|a|^2 a$. Therefore, the mean field equation (Eq(S.6)) reduces to the simpler form(Eq(S.14)).

$$\frac{\partial a}{\partial \xi} = (-\alpha + i\Delta\phi)a + ga^* - q|a|^2 a - i\frac{\beta_2}{2}\frac{\partial^2 a}{\partial t^2} \tag{S.14}$$

We introduce a small parameter ϵ. We express the signal field as $a = \sum_n \epsilon^n a_n$. The gain parameter is expanded about the threshold value at zero detuning as: $g = \alpha + p\epsilon^2$. We assume $(\Delta\phi - \frac{\beta_2}{2}\frac{\partial^2}{\partial t^2}) = \epsilon\Phi$. We introduce the slow time scale: $T = \epsilon^2\xi$. Substituting these in Eq(S.14), segregating terms with different orders of ϵ, satisfying the solvability conditions in successive orders we obtain the order parameter equation/ real SHE as follows:

$O(\epsilon)$: $-a_1 + a_1^* = 0$. This implies to the first order the signal field is a real quantity.

$O(\epsilon^2)$: $-\alpha a_2 + \alpha a_2^* + i\Phi a_1$=0

$O(\epsilon^3)$: $\frac{\partial a_1}{\partial T} = -\alpha a_3 + i\Phi a_2 + \alpha a_3^* + pa_1^* - q|a_1|^2 a_1$. Taking a conjugate of this and adding it to itself, we get: $\frac{\partial a_1}{\partial T} = pa_1 - qa_1^3 - \frac{(\Phi)^2}{2\alpha} a_1$.

We define $A = \epsilon a_1$. We get the resultant real SHE as:

$$\frac{\partial A}{\partial \xi} = (g - \alpha)A - qA^3 - \frac{1}{2\alpha}\left(\Delta\phi - \frac{\beta_2}{2}\frac{\partial^2}{\partial t^2}\right)^2 A \tag{S.15}$$

9 Nonlinear Bifurcation VS Phase Transition

9.1 Bifurcation Picture

Mathematically, our spectral phase transition can be interpreted as some nonlinear bifurcation phenomenon. However, it will only capture the spectral splitting. In the process, we loose several insights, that would otherwise be obtained in case we draw a comprehensive analogy with phase transitions, in particular by representing it as a case of dissipative/ non-equilibrium phase transition. These additional insights which cannot be captured by a nonlinear bifurcation description includes: order-disorder transition, the distinct phase noise properties in the degenerate and non-degenerate regimes, the power law scaling of the phase difference diffusion in the dis-ordered regime. Nevertheless, the presented spectral phase transition is in stark contrast with intensity dependent nonlinear bifurcation/ bifurcation of the homogeneous solution that is ubiquitous in Kerr nonlinear medium. The reasons are the following:

- One can take a mathematical route , overlooking the underlying physics and try to explain the spectral phase transition with nonlinear bifurcations at threshold. For example, a laser phase transition that happens at the threshold can be modelled as a bifurcation phenomenon and is a topic in standard textbooks [3], but such a description does not capture some of the essential behaviors of this transition [4]. We mention it for the sake of completeness, but we believe that this is not a complete description of the phenomenon.

  The bifurcation analysis of OPOs at degeneracy can be found in [5] which shows a pitch-fork bifurcation leading to binary phase states. For the non-degenerate regime, our analysis follows here. Bifurcation analysis of infinite-dimensional systems governed by PDE falls under the purview of center manifold theorem. We adopt the approach presented in [6]. The simplified version of mean-field dynamics governing the doubly resonant OPO evolution can be given by:

$$\frac{\partial a}{\partial \xi} = -\alpha a + ga^* + \mathrm{i}\Delta\phi a - \mathrm{i}\beta_2\frac{\partial^2 a}{\partial t^2} - g_s|a|^2 a \tag{S.16}$$

  where, all parameters stand for quantities as described in the main text, and g_s represents the gain saturation parameter. The homogeneous solution governed by Eq S.16 which corresponds to the degenerate spectrum is obtained by putting all the derivatives to zero. We thus obtain the steady-state relation as:

$$g^2 = (g_s I + \alpha)^2 + (\Delta\phi)^2 \tag{S.17}$$

  where we assume, $I = |a|^2$. The below threshold solution (trivial solution) is given by $I = 0$. Let's decompose the complex field (a) into real and imaginary components, i.e. $a = x + \mathrm{i}y$. For studying the bifurcation analysis of the fixed points of Eq (S.16), we put the derivative with respect to the slow time $\frac{\partial a}{\partial \xi} = 0$. We thus obtain the following set of equations:

$$\beta_2 \frac{\partial^2 x}{\partial t^2} = -\alpha y + \Delta\phi x - g y - g_s I y \quad \text{(S.18a)}$$

$$-\beta_2 \frac{\partial^2 y}{\partial t^2} = -\alpha x - \Delta\phi y + g x - g_s (I) x \quad \text{(S.18b)}$$

where, $I = |a|^2 = x^2 + y^2$. Let's define: $X = \frac{\partial x}{\partial t}$, and $Y = \frac{\partial y}{\partial t}$. Thus we obtain:

$$\dot{V} = JV \quad \text{(S.19a)}$$

where $V = [x \ X \ y \ Y]^T$, and J is the Jacobian which is given as

$$J = \begin{bmatrix} 0 & 1 & 0 & 0 \\ \frac{\Delta\phi}{\beta_2} & 0 & -\frac{\alpha+g+g_s I}{\beta_2} & 0 \\ 0 & 0 & 0 & 1 \\ \frac{\alpha-g+g_s I}{\beta_2} & 0 & \frac{\Delta\phi}{\beta_2} & 0 \end{bmatrix} \quad \text{(S.19b)}$$

The characteristic polynomial is then given by:

$$\lambda^4 \beta_2^2 - 2\lambda^2 \beta_2 \Delta\phi + (g_s I + \alpha)^2 + (\Delta\phi)^2 - g^2 = 0 \quad \text{(S.20)}$$

Let's consider the anomalous dispersion regime $\beta_2 < 0$. At the threshold ($g = \alpha$), we analyse the stability of the trivial solution ($I = 0$). From Eq S.20 we have: $(\lambda^2 + \Delta\phi)^2 = 0$. The repeated double eigenvalues are $\lambda = \pm \mathrm{i}\Delta\phi$. Therefore, we have a Hopf bifurcation at threshold when $\Delta\phi > 0$ in the anomalous dispersion regime. This Hopf bifurcation is related to the appearance of the non-degenerate oscillation. Similarly, one can analyse the normal dispersion regime ($\beta_2 > 0$) and observe Hopf bifurcation for $\Delta\phi < 0$ corresponding to the appearance of non-degenerate oscillation. At $\Delta\phi = 0$ all eignenvalues are zero in both cases.

This Hopf bifurcation is responsible for the instability of the trivial homogeneous steady state above threshold. Non-degenerate oscillatory solution emerges akin to roll patterns in spatially extended systems [7]. This analogy follows because the spectral phase transition is also governed by the universal Swift-Hohenberg equation. We can express the resultant OPO field as: $A = \Psi e^{\mathrm{i}\delta\omega\tau} + \Psi^* e^{-\mathrm{i}\delta\omega\tau}$. The linear stability analysis of this solution is performed in Eq(2) of the main text. Analysis of the accompanying nonlinear amplitude equation can also show that the OPO field in the non-degenerate regime is stable [7, 8].

- Intensity dependent nonlinear bifurcation arises when the homogeneous solution loses stability and the system switches to another stable branch. So intensity buildup is a pre-requisite for these kind of nonlinear instability. In our case, the OPO decide to oscillate in either degenerate or non-degenerate mode based on the gain competition which is a multi-mode co-operative effect. This OPO spectral selection happens in the growth stage before the onset of the gain saturation, and as we discussed earlier can even occur below the oscillation threshold in the form of a quantum image where the gain saturation is not present. Thus, gain saturation nonlinearity is not responsible for the bifurcation in case of spectral phase transition, rather it helps to quench the exponential growth.

- In systems exhibiting intensity dependent nonlinear bifurcation, the homogeneous solution loses stability beyond a critical value of a system parameter following which a new stable branch is adopted by the system. Thus, a buildup of a homogeneous solution is required for the onset of inhomogeneous solution. At the critical point the stability is exchanged. So, if we think in similar terms to explain our case, we have to make the association that when we transit from degenerate to non-degenerate regime by increasing the detuning (in anomalous dispersion) the degenerate OPO solution loses stability and the non-degenerate OPO solution is the new stable branch. But, in our case the OPO operating in the non-degenerate regime is not a result of the degenerate solution appearing first, and owing to its lack of stability the non-degenerate solution appears. This is in stark contrast to the familiar intensity dependent nonlinear bifurcation where a inhomogeneous solution cannot exist without the prior buildup the homogeneous solution. This can be also corroborated by the fact that spectral phase transition can be accessed by scanning the detuning in both directions, i.e. increasing the cavity length or by decreasing it as shown in Fig S.6.

9.2 Phase Transition Picture

We call it a spectral phase transition because the spectral behaviors of doubly resonant OPO bear a lot of properties akin to second-order phase transitions.

- **Order-Disorder transition and Symmetry Breaking:** In a degenerate OPO, the signal assumes bi-phase states, with two possible phases dictated by the pump. The two phase states are π separated. This property allows a degenerate OPO to emulate the Ising spin [9]. On the other hand, a non-degenerate OPO has a continuous U(1) degree of freedom. Classically, the sum of the phases of the signal and idler is dictated by the pump, but the difference of the phases is undetermined. This enables a non-degenerate OPO to emulate a XY spin configuration instead [10]. Thus, as we transit from the non-degenerate regime to the degenerate regime the U(1) symmetry is broken. So, we can associate degenerate OPO regime to an ordered phase and the non-degenerate OPO regime to a dis-ordered phase. Hence, at the critical point of the spectral phase transition, spontaneous symmetry breaking takes place.
- **Distinct Phase Noise Properties in the two regimes:** While both the degenerate and the non-degenerate regimes of a doubly resonant OPO are coherent, yet they display distinct phase noise properties [11]. The degenerate OPO is a phase locked sub-harmonic of the pump. Pump phase modulation can be transferred to the signal in an OPO operating at degeneracy. The linewidth in the degenerate regime is limited to the pump laser linewidth. On the other hand, in the non-degenerate regime the signal and idler are mutually coherent with the pump and phase anti-correlated. Away from the degeneracy, the signal and idler phase diffuse randomly. This phase diffusion is analogous to the phase diffusion observed in lasers that is responsible for the Schawlow-Townes limit. However, even in the non-degenerate OPO regime, the phase sum and the intensity difference of the twin beams are squeezed state observables [12].
- **Correlation properties and phase difference diffusion in the "disordered" case i.e. the non-degenerate regime:** Second-order phase transitions when characterized in terms of correlation functions exhibit power law dependence as a function of distance from the critical point, which represents universal scaling law for particular classes of continuous phase transitions. We consider the correlation spectrum of doubly resonant non-degenerate OPO in the presence of detuning. It is well known that twin beams displays squeezing in intensity difference and phase sum observable [12, 13]. Here, we investigate the correlation spectrum for the phase difference operator as a function of detuning in the non-degenerate (dis-ordered) regime. Our analysis reveals similar power law dependence as a function of detuning. This analogy reveals deep connections

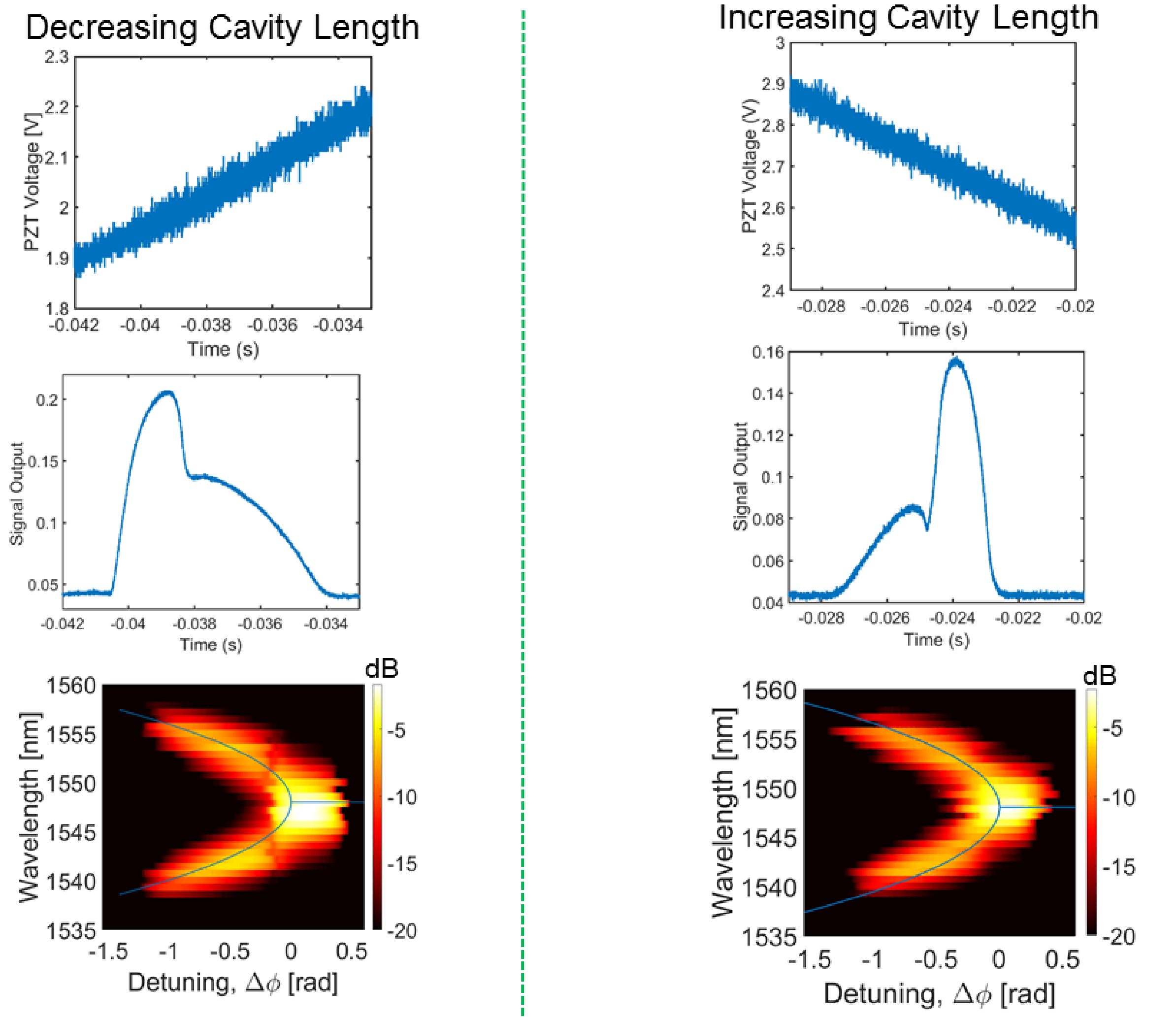

FIG S.6: Spectral phase transition accessed by detuning the cavity in both ways. The cavity dispersion is normal in this case. Note that the response of the slow detector looks different because of the slight difference in pump power in the two sets of measurements. The piezo tuning of the cavity is much slower compared to the lifetime of the intra-cavity field. This shows that non-degenerate regime can be accessed prior to degenerate regime and vice-versa.

between OPO spectral transition considered in our work and physics of phase transition.

Here, we investigate the correlation spectrum for the phase difference operator as a function of detuning in the non-degenerate (dis-ordered) regime.

The evolution of the signal (a_1), idler (a_2), and the pump (b) considering the vacuum fluctuations associated with the dissipations (intrinsic loss and out-coupling loss) is given by:

$$\dot{a}_1 = -\alpha a_1 + \mathrm{i}\Delta a_1 + gba_2^* + \sqrt{2\gamma}V_{\gamma,1} + \sqrt{2\mu}V_{\mu,1} \tag{S.21a}$$

$$\dot{a}_2 = -\alpha a_2 + \mathrm{i}\Delta a_2 + gba_1^* + \sqrt{2\gamma}V_{\gamma,2} + \sqrt{2\mu}V_{\mu,2} \tag{S.21b}$$

$$\dot{b} = -\alpha_0 b - ga_1a_2 + \sqrt{2\gamma_0}V_{\gamma,0} + \sqrt{2\mu_0}V_{\mu,0} + \sqrt{2\mu_0}b_0^{\mathrm{in}} \tag{S.21c}$$

Here, we assume identical loss parameters for the signal and idler. α (total loss) $=\gamma+\mu$, with γ and μ representing the intrinsic and out-coupling loss respectively. The nonlinear co-efficient is denoted by g, and detuning is symbolized by Δ. We will perform adiabatic elimination of the pump, which has drastically different decay lifetime, which in the asymptotic limit represents the non-resonant pumping case. The pump parameters are denoted with subscript 0. The coherent pump driving the OPO above threshold is given by b_0^{in}. The Langevin forces V representing the fluctuations have zero mean-field and obey the following correlation properties:
$\left\langle V_{s,l}(\omega)V^{\dagger}_{s',l'}(\omega')\right\rangle = \delta_{ss'}\delta_{l,l'}\delta(\omega-\omega'), \left\langle V^{\dagger}_{s,l}(\omega)V_{s',l'}(\omega')\right\rangle = \left\langle V_{s,l}(\omega)V_{s',l'}(\omega')\right\rangle = \left\langle V^{\dagger}_{s,l}(\omega)V^{\dagger}_{s',l'}(\omega')\right\rangle = 0.$

Considering only the mean-field, Eq (8), reduces to the follwing:

$$\dot{a_1} + \alpha a_1 = \mathrm{i}\Delta a_1 + gba_2^* \tag{S.22a}$$

$$\dot{a_2} + \alpha a_2 = \mathrm{i}\Delta a_2 + gba_1^* \tag{S.22b}$$

$$\dot{b} + \alpha_0 b = \sqrt{2\mu_0}b_0^{\mathrm{in}} - ga_1a_2 \tag{S.22c}$$

Clearly, $|b|^2 = \frac{\alpha^2+\Delta^2}{g^2}$ has to be satisfied. If we choose the driving pump field phase appropriately such that the steady-state intra-cavity pump field is given by, $b = \frac{\alpha-\mathrm{i}\Delta}{g}$, then we can assume the signal and idler fields to be equal and real quantities.

The fluctuations in the pump field is given by:

$$\dot{\delta b} = -\alpha_0\delta b - g(a_1\delta a_2 + a_2\delta a_1) + \sqrt{2\gamma_0}V_{\gamma,0} + \sqrt{2\mu_0}V_{\mu,0} \tag{S.23a}$$

Performing adiabatic elimination we get,

$$\delta b = -\frac{g}{\alpha_0}(a_1\delta a_2 + a_2\delta a_1) + \frac{\sqrt{2\gamma_0}}{\alpha_0}V_{\gamma,0} + \frac{\sqrt{2\mu_0}}{\alpha_0}V_{\mu,0} \tag{S.23b}$$

The fluctuations in the signal and idler field are governed by:

$$\dot{\delta a_1} = -(\alpha - \mathrm{i}\Delta)\delta a_1 + gb\delta a_2^* + ga_2^*\delta b + \sqrt{2\gamma}V_{\gamma,1} + \sqrt{2\mu}V_{\mu,1} \tag{S.24a}$$

$$\dot{\delta a_2} = -(\alpha - \mathrm{i}\Delta)\delta a_2 + gb\delta a_1^* + g a_1^* \delta b + \sqrt{2\gamma}V_{\gamma,2} + \sqrt{2\mu}V_{\mu,2} \tag{S.24b}$$

We substitute the expression for pump fluctuations obtained in Eq (S.23 b) in Eq (S.24) to obtain the evolution of required observables. We define: $\delta p = \frac{1}{\sqrt{2}}(\delta a_1 - \delta a_2 + \delta a_1^* - \delta a_2^*)$ which stands for the amplitude fluctuations difference operator for the twin beam, and $\delta q = \frac{1}{\sqrt{2}}(-\mathrm{i}(\delta a_1 - \delta a_2) + \mathrm{i}(\delta a_1^* - \delta a_2^*))$ which represents the phase difference fluctuations operator.

The evolution of these observables is governed by:

$$\begin{bmatrix}\dot{\delta q}\\ \delta p\end{bmatrix} = J\begin{bmatrix}\delta q\\ \delta p\end{bmatrix} + \sqrt{2\gamma}\begin{bmatrix}W_{\gamma,\frac{\pi}{2}}\\ W_{\gamma,0}\end{bmatrix} + \sqrt{2\mu}\begin{bmatrix}W_{\mu,\frac{\pi}{2}}\\ W_{\mu,0}\end{bmatrix} \tag{S.25a}$$

where, $J = \begin{bmatrix}0 & -2\Delta\\ 0 & -2\alpha\end{bmatrix}$. $W_{s,\frac{\pi}{2}} = \frac{1}{\sqrt{2}}[-\mathrm{i}(V_{s,1} - V_{s,2}) + h.c]$, $W_{s,0} = \frac{1}{\sqrt{2}}[(V_{s,1} - V_{s,2}) + h.c]$. $h.c$ stands for Hermitian conjugate. It is interesting to observe that these observables are decoupled from the pump noise.

The input-output relation which relates the intra-cavity field to the extracted field is given by:

$$\begin{bmatrix}\delta Q_{\mathrm{out}}\\ \delta P_{\mathrm{out}}\end{bmatrix} = \sqrt{2\mu}\begin{bmatrix}\delta q\\ \delta p\end{bmatrix} - \begin{bmatrix}W_{\mu,\frac{\pi}{2}}\\ W_{\mu,0}\end{bmatrix} \tag{S.25b}$$

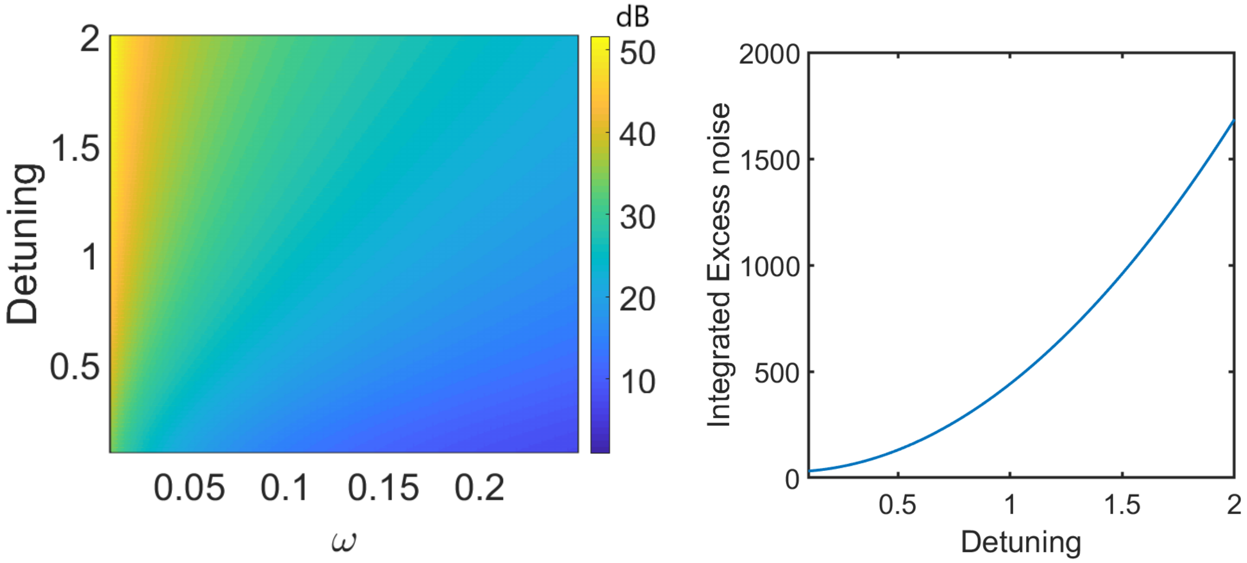

(a) (b)

FIG S.7: Noise in the phase difference operator in the non-degenerate regime. a) The phase difference noise spectrum is plotted as a function of detuning, showing signatures of phase diffusion. b) Integrated excess noise in arbitrary units as a function of detuning of the band-limited phase-difference quadrature signal. The integrated noise follows a power law behaviour with detuning as given by Eq (S.28b).

In the Fourier domain this can be expressed as:

$$\begin{bmatrix}\delta Q_{\mathrm{out}}(\omega)\\ \delta P_{\mathrm{out}}(\omega)\end{bmatrix} = -\sqrt{2\mu}[J + \mathrm{i}\omega I_2]^{-1}\left(\sqrt{2\gamma}\begin{bmatrix}W_{\gamma,\frac{\pi}{2}}\\ W_{\gamma,0}\end{bmatrix} + \sqrt{2\mu}\begin{bmatrix}W_{\mu,\frac{\pi}{2}}\\ W_{\mu,0}\end{bmatrix}\right) - \begin{bmatrix}W_{\mu,\frac{\pi}{2}}\\ W_{\mu,0}\end{bmatrix} \tag{S.26}$$

We can define the output correlation matrix as: $C^{\text{out}}(\omega) =_{-\infty}^{\infty} d\omega' \left\langle \begin{bmatrix} \delta Q_{\text{out}}(\omega) \\ \delta P_{\text{out}}(\omega) \end{bmatrix} \begin{bmatrix} \delta Q_{\text{out}}(\omega) \\ \delta P_{\text{out}}(\omega) \end{bmatrix}^T \right\rangle$

Therefore, the correlation spectrum is given by the following expression:

$$\begin{aligned} C^{\text{out}}(\omega) &= \{2\alpha\rho(J + \mathrm{i}\omega I_2)^{-1} + I_2\} C^{\text{in}}(\omega) \{2\alpha\rho(J - \mathrm{i}\omega I_2)^{-1} + I_2\}^T \\ &+ 4\alpha^2\rho(1-\rho)\{(J + \mathrm{i}\omega I_2)^{-1}\} C^{\text{in}}(\omega) \{(J - \mathrm{i}\omega I_2)^{-1}\}^T \end{aligned} \tag{S.27}$$

where, $C^{\text{in}}(\omega) = \begin{bmatrix} 1 & \mathrm{i} \\ -\mathrm{i} & 1 \end{bmatrix}$ is the input correlation matrix, and I_2 is an identity matrix of second order.

We are interested in the spectrum for phase diffusion difference operator $S_q(\omega)$. This can be extracted from the output correlation matrix, $S_q(\omega) = C_{11}^{\text{out}}(\omega)$.

Thus, we obtain the familiar expression for the phase diffusion in the absence of detuning [12]:

$$S_q(\omega) = 1 + \frac{4\rho\alpha^2}{\omega^2} \tag{S.28a}$$

where, $\rho = \frac{\mu}{\alpha}$.

In the presence of detuning we derive the general expression:

$$S(\omega) = 1 + \frac{4\rho\alpha^2}{\omega^2}\left(1 + \frac{4\Delta^2}{\omega^2 + 4\alpha^2}\right) \tag{S.28b}$$

Now, if we measure the excess noise (above shot-noise) for the phase difference quadrature (δq), after band pass filtering the signal, we obtain a power law dependence as a function of detuning (i.e. distance from the critical point). The results are shown in Fig S.7.

- **Lyapunov Functional as Free Energy:** In our case, we can define a Lyapunov functional which plays a role similar to free-energy in equilibrium phase transitions[14]. The Swift-Hohenberg equation given by (S.15) can also be expressed in a variational structure as:

$$\frac{\partial A}{\partial \xi} = -\frac{\partial F}{\partial A} \tag{S.29a}$$

where, the Lyapunov Functional is $F[A(t,\xi)]$. F is given by:

$$F =_{-\frac{T_R}{2}}^{\frac{T_R}{2}} dt \left(-\frac{1}{2}(g-\alpha)A^2 + \frac{q}{4}A^4 + \frac{1}{2\alpha}\left[(\Delta\phi - \frac{\beta_2}{2}\frac{\partial^2}{\partial t^2})A \right]^2 \right) \tag{S.29b}$$

where, T_R is the round-trip time. All other parameters are defined in Section 8 of the supplementary information. It follows that,

$$\frac{dF}{d\xi} = -\left(\frac{\partial A}{\partial \xi}\right)^2 \leq 0 \tag{S.29c}$$

Hence, $\frac{dF}{d\xi} < 0$ provided $\frac{\partial A}{\partial \xi} \neq 0$.

Thus, with appropriate boundary conditions all solutions evolve towards the stationary state of the energy landscape defined by F. $F[A]$ acts as the free-energy of the ystem. Stable (unstable) solutions correspond to local minima (maxima) of this free-energy like entity.

10 Quantum Image of the Spectral Phase Transition below threshold

The spectral phase transitions that we discussed as a near threshold phenomenon (above threshold) also leaves a quantum image below threshold [15]. In our case, it is manifested in terms of the quadrature squeezing spectrum while we employ local oscillator shaping. Our system is essentially a multi-mode system (covering both degenerate and non-degenerate oscillating longitudinal modes). So, to completely characterize the squeezing behaviour of such an OPO below threshold, we need to spectrally shape the local oscillator. Temporal mode shaping of the local oscillator according to the super-modes for a synchronously pumped OPO is also used [16]. Thus, we investigate the squeezing spectrum obtained by continuous spectral tuning of the local oscillator (LO), when the OPO is below threshold. The quantum image of the spectral phase transition is generated below threshold. The simulation result is shown in Fig S.8(a) . Clearly, at a finite detuning, the maximum squeezing can be measured by a non-degenerate LO, that follows the spectral splitting according to the spectral phase transition happening above threshold. Thus, the optimum LO, will extract better squeezing than a degenerate LO as shown in Fig S.8(b).

Let the local oscillator be expressed as $LO = P\mathrm{e}^{\mathrm{i}m\tau} + P\mathrm{e}^{-\mathrm{i}m\tau}$. The quadratures projected by the LO are given by: $q_0 = \frac{1}{\sqrt{2}}\left[(a_s\mathrm{e}^{-\mathrm{i}m\tau} - a_i\mathrm{e}^{\mathrm{i}m\tau}) + (h.c)\right]$ and $q_{\frac{\pi}{2}} = \frac{1}{\sqrt{2}}\left[-\mathrm{i}(a_s\mathrm{e}^{-\mathrm{i}m\tau} - a_i\mathrm{e}^{\mathrm{i}m\tau}) + (h.c)\right]$. The evolution of the fluctuation associated with a_s and a_i are given by:

$$\dot{a_s} = -\alpha a_s + g a_i^* + \mathrm{i}\Delta a_s - \mathrm{i}\frac{\beta_2}{2}\frac{\partial^2 a_s}{\partial \tau^2} + \sqrt{2\gamma}V_{\gamma,s} + \sqrt{2\mu}V_{\mu,s} \tag{S.30a}$$

$$\dot{a_i} = -\alpha a_i + g a_s^* + \mathrm{i}\Delta a_i - \mathrm{i}\frac{\beta_2}{2}\frac{\partial^2 a_i}{\partial \tau^2} + \sqrt{2\gamma}V_{\gamma,i} + \sqrt{2\mu}V_{\mu,i} \tag{S.30b}$$

Here, we assume identical loss parameters for the signal and idler. α (total loss) $=\gamma + \mu$, with γ and μ representing the intrinsic and out-coupling loss respectively. The parametric gain is denoted by g, and detuning is symbolized by Δ. The Langevin forces V representing the fluctuations (due to out-coupling and intrinsic loss) have zero mean-field and obey the following correlation properties: $\left\langle V_{s,l}(\omega)V^{\dagger}_{s',l'}(\omega')\right\rangle = \delta_{ss'}\delta_{l,l'}\delta(\omega - \omega')$, $\left\langle V^{\dagger}_{s,l}(\omega)V_{s',l'}(\omega')\right\rangle = \left\langle V_{s,l}(\omega)V_{s',l'}(\omega')\right\rangle = \left\langle V^{\dagger}_{s,l}(\omega)V^{\dagger}_{s',l'}(\omega')\right\rangle = 0$.

The evolution of the quadratures is governed by:

$$\dot{\begin{bmatrix} q_0 \\ q_{\frac{\pi}{2}} \end{bmatrix}} = J\begin{bmatrix} q_0 \\ q_{\frac{\pi}{2}} \end{bmatrix} + \sqrt{2\gamma}\begin{bmatrix} W_{\gamma,\frac{\pi}{2}} \\ W_{\gamma,0} \end{bmatrix} + \sqrt{2\mu}\begin{bmatrix} W_{\mu,\frac{\pi}{2}} \\ W_{\mu,0} \end{bmatrix} \tag{S.31a}$$

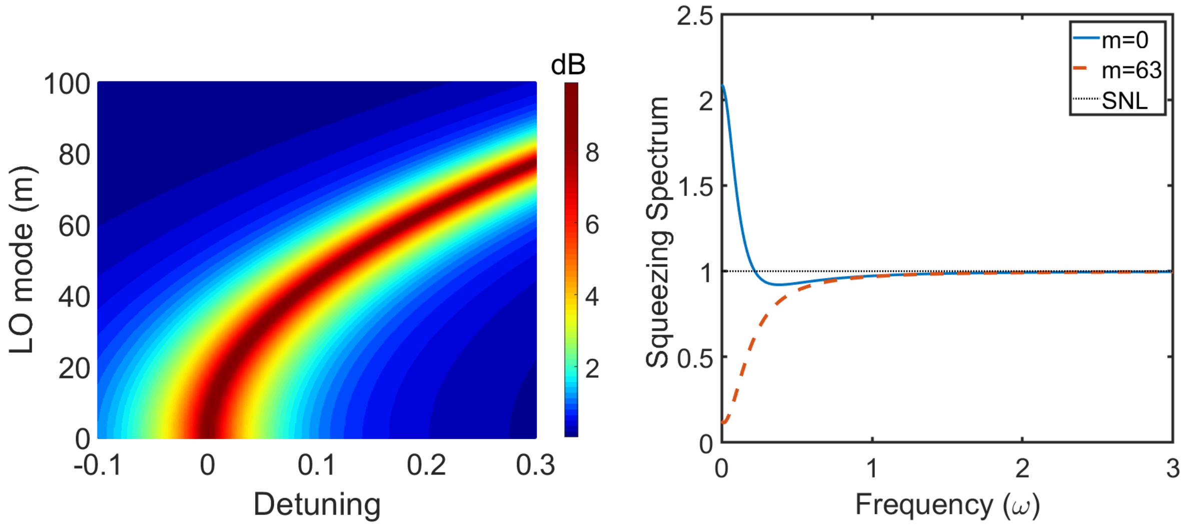

(a) (b)

FIG S.8: Quantum image of the spectral phase transition below threshold. a) Maximum squeezing can be extracted by continuous spectral shaping of the LO following the spectral splitting as it appears in the spectral phase transition. b) Squeezing spectrum obtained using the optimum LO (m=63), compared to an un-optimized LO (m=0).

where, $J = \begin{bmatrix} -\alpha - g & \Delta + \frac{\beta_2}{2}m^2 \\ \Delta + \frac{\beta_2}{2}m^2 & \alpha + g \end{bmatrix}$. $W_{s,\frac{\pi}{2}} = \frac{1}{\sqrt{2}}[-\mathrm{i}(V_{s,1} - V_{s,2}) + h.c]$, $W_{s,0} = \frac{1}{\sqrt{2}}[(V_{s,1} - V_{s,2}) + h.c]$. $h.c$ stands for Hermitian conjugate.

The input-output relation which relates the intra-cavity field to the extracted field is given by:

$$\begin{bmatrix} Q_0^{\rm out} \\ Q_{\frac{\pi}{2}}^{\rm out} \end{bmatrix} = \sqrt{2\mu} \begin{bmatrix} q_0 \\ q_{\frac{\pi}{2}} \end{bmatrix} - \begin{bmatrix} W_{\mu,\frac{\pi}{2}} \\ W_{\mu,0} \end{bmatrix} \tag{S.31b}$$

In the Fourier domain this can be expressed as:

$$\begin{bmatrix} Q_0^{\rm out}(\omega) \\ Q_{\frac{\pi}{2}}^{\rm out}(\omega) \end{bmatrix} = -\sqrt{2\mu}[J + \mathrm{i}\omega I_2]^{-1} \left(\sqrt{2\gamma} \begin{bmatrix} W_{\gamma,\frac{\pi}{2}} \\ W_{\gamma,0} \end{bmatrix} + \sqrt{2\mu} \begin{bmatrix} W_{\mu,\frac{\pi}{2}} \\ W_{\mu,0} \end{bmatrix} \right) - \begin{bmatrix} W_{\mu,\frac{\pi}{2}} \\ W_{\mu,0} \end{bmatrix} \tag{S.32}$$

We can define the output correlation matrix as: $C^{\rm out}(\omega) =_{-\infty}^{\infty} d\omega' \left\langle \begin{bmatrix} Q_0^{\rm out}(\omega) \\ Q_{\frac{\pi}{2}}^{\rm out}(\omega) \end{bmatrix} \begin{bmatrix} Q_0^{\rm out}(\omega) \\ Q_{\frac{\pi}{2}}^{\rm out}(\omega) \end{bmatrix}^T \right\rangle$

Therefore, the correlation spectrum is given by the following expression:

$$\begin{aligned} C^{\rm out}(\omega) = \{2\alpha\rho(J + \mathrm{i}\omega I_2)^{-1} + I_2\} C^{\rm in}(\omega) \{2\alpha\rho(J - \mathrm{i}\omega I_2)^{-1} + I_2\}^T \\ + 4\alpha^2\rho(1-\rho)\{(J + \mathrm{i}\omega I_2)^{-1}\} C^{\rm in}(\omega) \{(J - \mathrm{i}\omega I_2)^{-1}\}^T \end{aligned}$$

(S.33)

where, $C^{\text{in}}(\omega) = \begin{bmatrix} 1 & \mathrm{i} \\ -\mathrm{i} & 1 \end{bmatrix}$ is the input correlation matrix, and I_2 is an identity matrix of second order.

We are interested in the spectrum for the squeezed quadrature $S_{q_0}(\omega)$. This can be extracted from the output correlation matrix, $S_{q_0}(\omega) = C_{11}^{\text{out}}(\omega)$.

11 Effect of Pump power ($p = \frac{b}{b_0}$):

Here, we numerically investigate the dependence of oscillation range (degenerate and non-degenerate) on the pump power (expressed in terms of number of times above threshold).

As evident from Eq(5) of the main text the threshold in the degenerate regime is a function of detuning (Δ). Although, the mean field model incorrectly predicts a constant threshold in the non-degenerate regime, the CW gain theory described in section 3 reveal that the threshold is a function of detuning. Here we study the effect of p on the OPO spectrum.

In the near threshold regime of operation we can neglect back-conversion from the signal to the pump. But at several times above threshold one cannot neglect the effect of the back-conversion which is caused by second harmonic generation of the signal. If we assume that there is no linear loss (attenuation in fibers, insertion loss of components, etc) then energy conservation suggests that the combined power of the signal and pump should remain invariant. The signal derives energy from the pump depletion as shown in Fig. S.10.

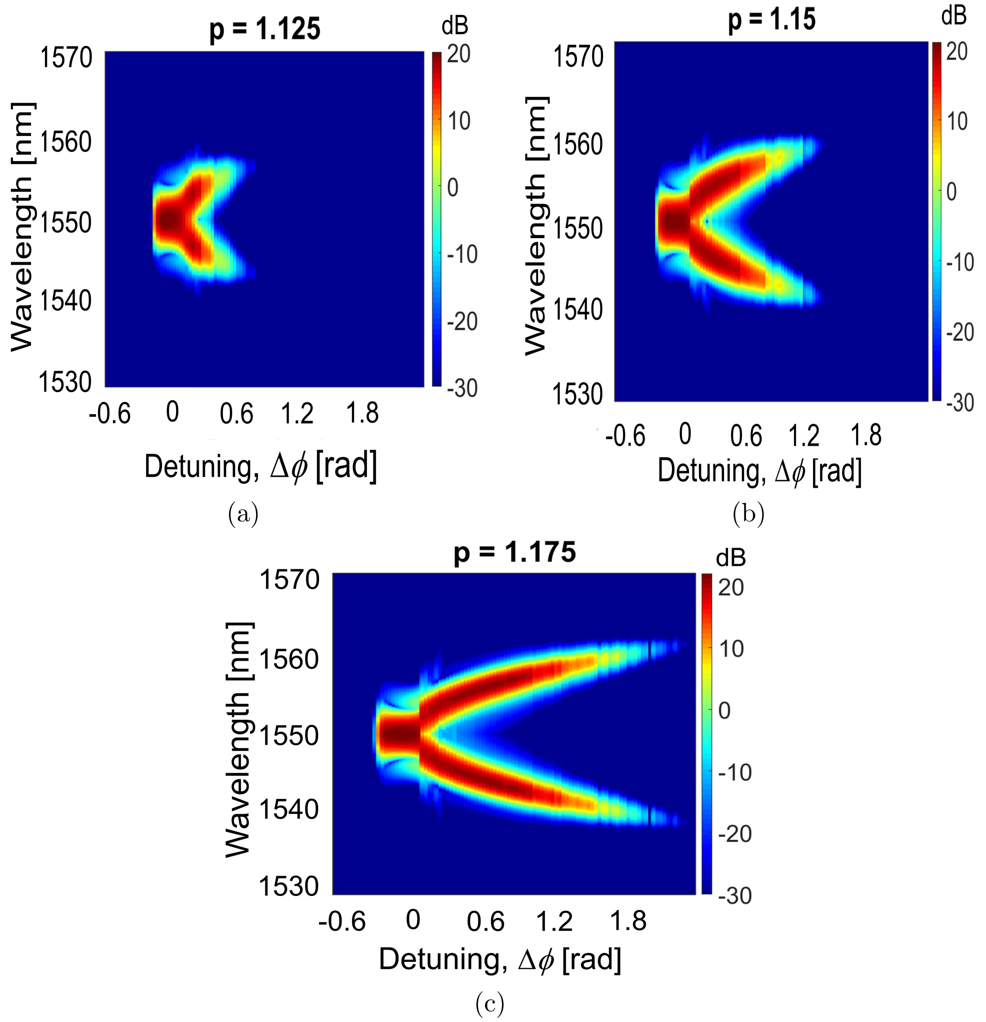

Figure S.9: Power dependence of the spectral phase transition. Plots of the spectrum for different values of the normalized pump amplitude: a) p= 1.125, b) p= 1.15, and c) p=1.175.

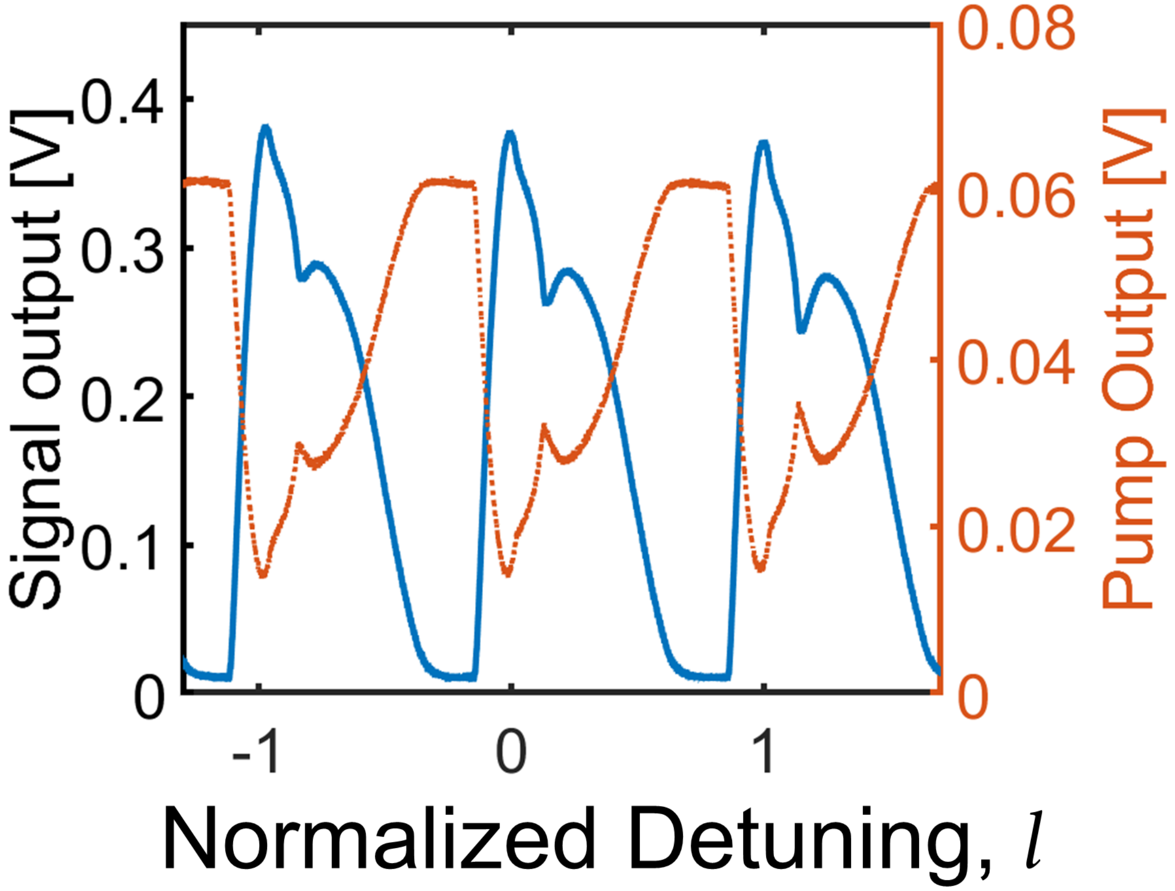

Figure S.10: Measured signal and pump power showing pump depletion. The pump is non-resonant and follows the same dependence with the cavity detuning as that of the signal.

12 Time domain representation:

Here, we investigate how the OPO pulses appear in time domain representation both in degenerate and non-degenerate regimes of operation.

Here we present the temporal waveform of the pulses in the OPO. These results are obtained from the numerical simulation of the full spatio-temporal model. It is observed that in the degenerate regime, steady state signal pulses are obtained. While in the non-degenerate regime the signal and idler pulses interfere with each other, and produce an interference pattern.

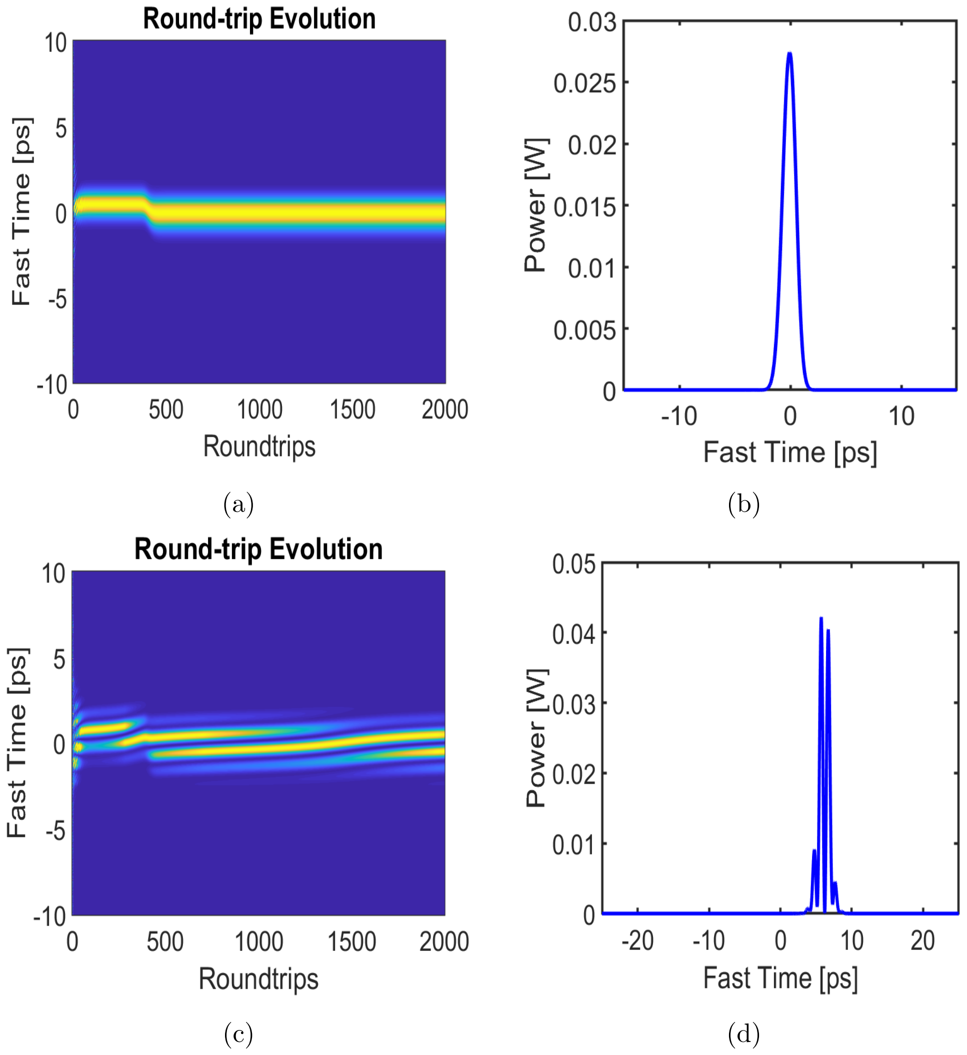

Figure S.11: Temporal representation of the OPO. a) Round-trip evolution of the signal emanating from noise in the degenerate limit. The signal at the end of each round-trip is normalized to the maximum value at that round-trip. b) Signal waveform in the degenerate case at the end of 2000 round-trips (steady-state reached).c) Round-trip evolution of the signal and idler emanating from noise in the non-degenerate limit. d) Signal/idler interference waveform in the non-degenerate case at the end of 2000 round-trips .

13 Frequency domain representation (Frequency Comb Picture):

If we consider a frequency comb picture of the OPO, then the transition in the non-degenerate regime will lead to the co-existence of signal and idler combs. Here, we investigate how the OPO behaves differently in the two regimes (degenerate and non-degenerate) in the frequency comb picture.

In the degenerate regime we have a single comb and there is no beatnote. However, there is an uncertainty of the carrier envelope phase offset frequency (f^s_{CEO}), which can be either $f^p_{CEO}/2$ or $f^p_{CEO}/2+f_{REP}/2$. This is related to the fact that there is a possibility of bi-phase states. In the non-degenerate regime two combs are formed, one corresponding to the signal and the other to the idler. Beatnote appears between the signal and idler. The beatnote increases with detuning, but it always satisfies the requirement that $f^s_{CEO} + f^i_{CEO} = f^p_{CEO}$ or $f^s_{CEO} + f^i_{CEO} = f^p_{CEO} + f_{REP}$.

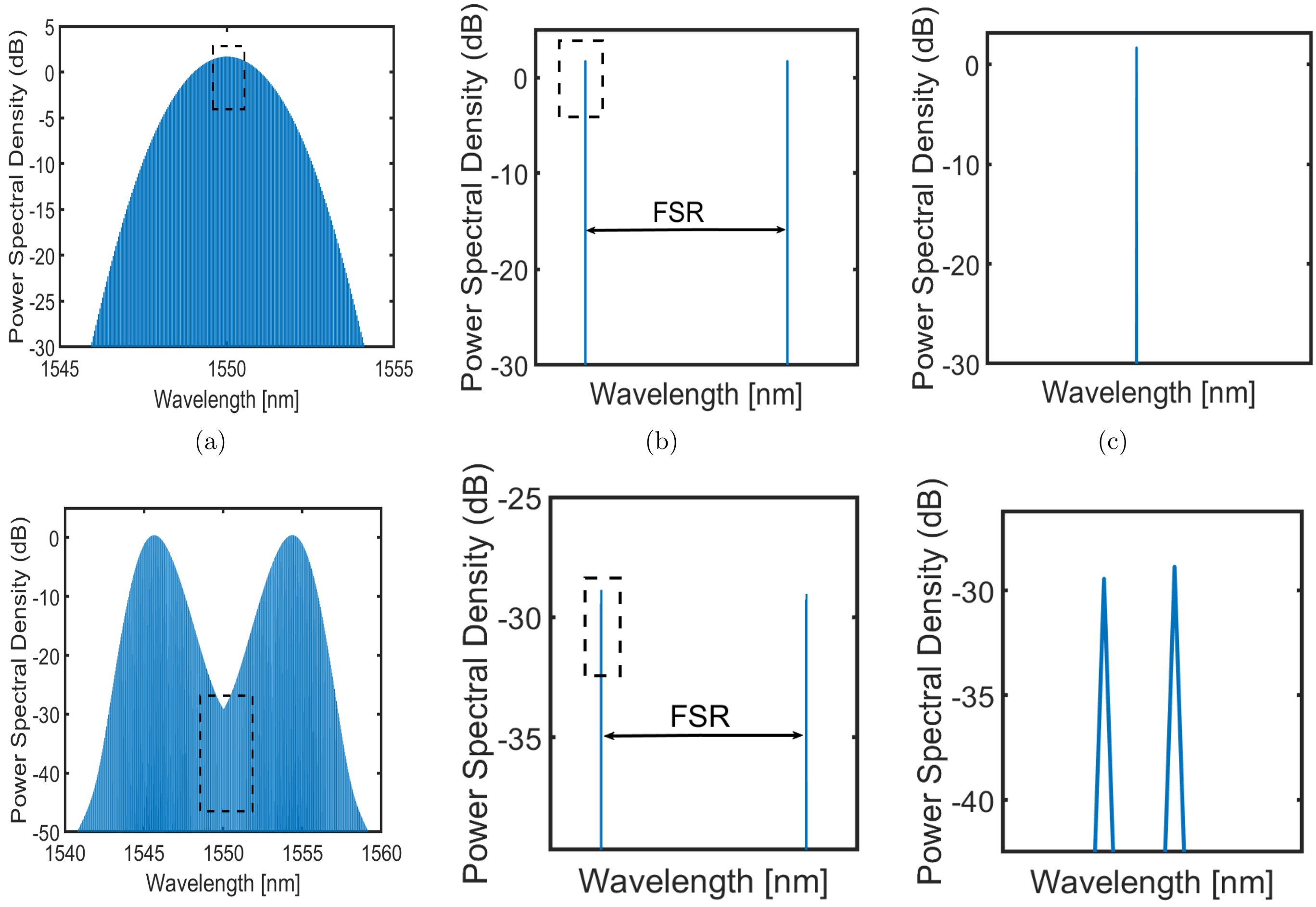

(a) (b) (c)

(d) (e) (f)

Figure S.12: Frequency domain representation of the OPO. a,b,c) Corresponds to the degenerate phase. d,e,f) corresponds to the non-degenerate phase. It clearly shows the presence of two interleaved combs, which results in a beatnote. The dotted box is zoomed in the following sub-figure.

14 Degenerate Optical Parametric Oscillator in Kerr nonlinear medium:

Phase sensitive phase conjugation process occurring in a Kerr nonlinear medium in the presence of two pumps can emulate the quadratic OPO behavior. We obtain the parametric gain associated with the

phase conjugation process in such a scenario.

We can realize degenerate parametric oscillation in Kerr medium if dual pumps are employed [17]. Let us assume we inject two CW pumps of equal power in a non-resonant Kerr nonlinear medium. Our analysis can be extended to resonant case easily. We represent pump 1 as $A_1 = \sqrt{P}e^{i(\phi_1(z)-\omega_1 t)}$, where $\omega_1 = \omega_0 - \Delta$, such that Δ is the detuning from the center of degeneracy (ω_0). Pump 2 is given by: $A_2 = \sqrt{P}e^{i(\phi_2(z)-\omega_2 t)}$, where $\omega_2 = \omega_0 + \Delta$.

If a small signal perturbation is considered, then it can undergo the following nonlinear interactions, namely modulation instability (MI), four wave mixing Bragg Scattering, and phase conjugation. These processes are depicted in Fig S.10. For parametric oscillation that mimics a second-order nonlinear down conversion process, we are interested in the phase conjugation interaction.

MI doesn't appear in the normal GVD regime, which is the dispersion of interest to observe spectral phase transition. However in the anomalous dispersion regime, the generated MI sidebands are also transferred by the Kerr interaction to higher order FWM (four-wave mixing) sidebands. This leads to a collective behavior of the instability [18, 19].

We consider a signal of the form: $A_s = B_s(z)e^{i(\phi_2(z)-\omega_s t)}$, where $\omega_s = \omega_0 + \delta\omega$, such that $\delta\omega$ is the detuning from the center of degeneracy (ω_0). Another idler counterpart is assumed to be: $A_i = B_i(z)e^{i(\phi_1(z)-\omega_i t)}$, where $\omega_i = \omega_0 - \delta\omega$. Clearly, $\delta\omega = 0$ implies degenerate parametric oscillation. The signal and idler are considered to be small signals such that it is amenable to linearization/ linear stability analysis.

The evolution of the optical fields in the non-resonant Kerr nonlinear medium is governed by the Schrodinger equation which is given by:

$$\frac{\partial A}{\partial z} = i\beta(i\frac{\partial}{\partial t})A + i\gamma|A|^2 A \tag{S.34}$$

where, $\beta(\omega)$ is the Taylor expansion of the dispersion function, incorporating all order of GVDs, and γ is the nonlinearity co-efficient. The total complex optical field at the input is given by: $A = A_1 + A_2 + A_i + A_s$. The leading order expansion in the undepleted pump approximation yields: $\phi_1(z) = \beta(\omega_1)z + \gamma(3P)z$, and $\phi_2(z) = \beta(\omega_2)z + \gamma(3P)z$.

The evolution of the signal and idler will be governed by:

$$\frac{d}{dz}\begin{bmatrix} B_i \\ B_s^* \end{bmatrix} = \begin{bmatrix} i[\beta(\omega_i) - \beta(\omega_1) + \gamma P] & i2\gamma P \\ -i2\gamma P & -i[\beta(\omega_s) - \beta(\omega_2) + \gamma P] \end{bmatrix} \begin{bmatrix} B_i \\ B_s^* \end{bmatrix} \tag{S.35}$$

Considering only second order GVD, we have $\beta(\omega_i) - \beta(\omega_1) = \beta(\omega_s) - \beta(\omega_2) = \frac{\beta_2}{2}\left((\delta\omega)^2 - \Delta^2\right)$. The larger eigenvalue of the linear stability matrix determines the gain and is given by:

$$\lambda = \sqrt{\{6\gamma P - \beta_2(\Delta^2 - (\delta\omega)^2)\}\{2\gamma P + \beta_2(\Delta^2 - (\delta\omega)^2)\}} \tag{S.36}$$

It should be noted that the complete dynamics of the dual-pump mediated interactions in a Kerr medium cannot be described by the incoherently coupled nonlinear Schrodinger equations which neglects the multiple FWM interactions [20].

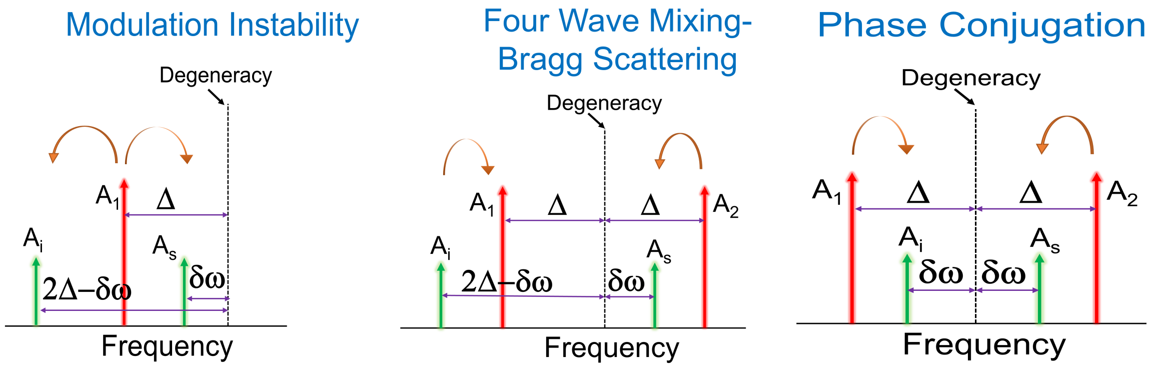

(a) (b) (c)

Figure S.13: Kerr nonlinearity with dual pump. Nonlinear interactions can be classified as: a) Modulation Instability b) Four-wave Mixing Bragg Scattering c) Phase Conjugation.

15 Phase behavior of OPO around the spectral phase transition:

In the degenerate regime, the phase of the signal is entrained to the pump phase and exhibits bi-phase states which is the underlying principle for the operation of OPO based optical Ising machines [9]. In the non-degenerate regime, the signal and idler becomes distinguishable and their individual phases can be random (not dictated by the pump), as long as the momentum conservation is satisfied. Here we observe that as the OPO undergoes a transition from the degenerate spectral regime to non-degenerate spectral regime around the critical point, the phase of the signal undergoes a drastic change. In the degenerate regime, the signal can assume only two possible phases, at a fixed cavity detuning and pump reference phase. Thus the degenerate case corresponds to a ordered state. While in the non-degenerate case the signal can assume any random phase and thus corresponds to a dis-ordered state. One can draw an analogy of this feature to optical ferro-magnetic phase (degenerate regime) and optical para-magnetic phase (non-degenerate regime) [21]. Thus the spectral phase transition also represents a order-disorder phase transition.

In order to show that in the degenerate regime, the absolute phase of the bi-phase states changes with detuning we adopt a simplified model of CW OPO operating at degeneracy. The predictions of this model match qualitatively with the results obtained from numerical simulation of pulsed synchronously pumped OPOs. The detuning of the cavity contributes an additional phase while determining the spontaneously selected OPO signal phase for maximum parametric gain. The equation governing the CW OPO operating at degeneracy is given by:

$$\frac{da}{d\xi} = -\alpha a + i\Delta\phi a + g a^* - g_s|a|^2 a \tag{S.37}$$

where a is the signal envelope, ξ is the slow time, α denotes the loss, $\Delta\phi$ is the cavity detuning. g represents the parametric gain and depends on the pump amplitude and phase, where we can assume the phase of the pump to be zero for reference, which renders g to be real. g_s stands for the gain saturation term. We assume the complex signal envelope to be: $a = Ae^{i\theta}$. Substituting this in Eq (S.37) and segregating the real and imaginary terms we get in the steady state:

$$0 = -\alpha + g\cos(2\theta) - g_s|A|^2 \tag{S.38a}$$

$$0 = \Delta\phi - g\sin(2\theta) \tag{S.38b}$$

From Eq (S.38a) we get, $|A|^2 = \frac{g\cos(2\theta)-\alpha}{g_s}$. Eq (S.38b) yields the detuning dependant bi-phase states as: $\theta = \frac{1}{2}\sin^{-1}\left(\frac{\Delta\phi}{g}\right)$ or $\theta = \pi + \frac{1}{2}\sin^{-1}\left(\frac{\Delta\phi}{g}\right)$. In the small detuning limit, we get $\theta \simeq \frac{\Delta\phi}{2g}$. Thus we see that the absolute value of the bi-phase state of the signal with respect to the pump phase reference varies linearly with detuning. This absolute phase varies inversely with g, i.e. the input pump power.

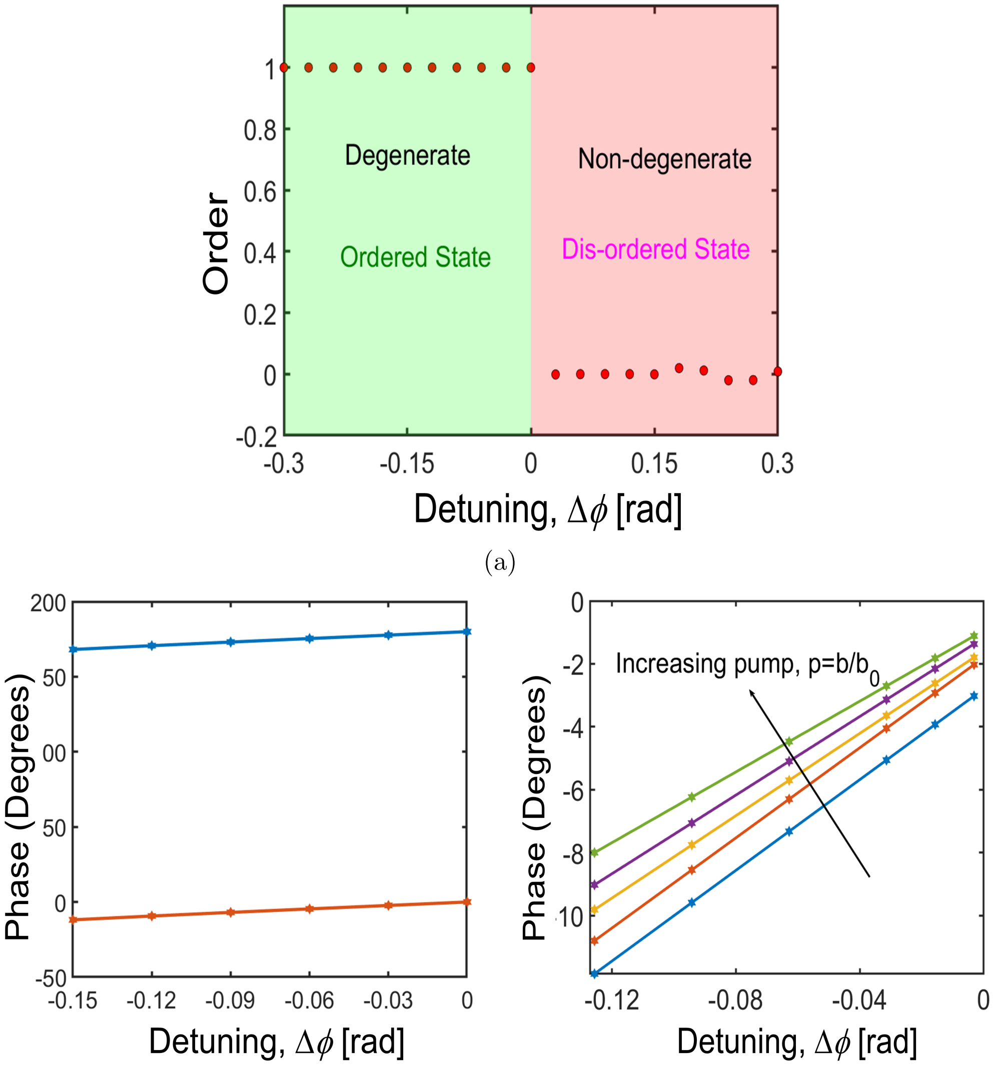

Figure S.14: Phase behavior of OPO. a) Order-disorder phase transition. The OPO is in an ordered phase state (bi-phase) in the degenerate regime, and dis-ordered phase state (random phase) in the non-degenerate regime. b) Existence of bi-phase state in the degenerate spectral regime. Here we have considered anomalous cavity dispersion. The absolute value of the signal phase change linearly with detuning. c) Absolute value of the signal phase in the degenerate regime for different input pump power values.

We define order function for the bi-phase state as $Order = \sum_i f(\frac{\theta_{i+1}-\theta_i}{\pi})$, where θ_j is the phase of the signal measured in radian occurring the j-th run. Every run of the OPO means that the signal phase will be spontaneously selected above threshold of parametric oscillation. The function f is defined as: $f(0) = f(1) = f(-1) = 1$, else $f(x) = x$.

16 Connection to PT symmetry:

Although, our system is a non-Hermitian system, the presented critical point should not be confused with an exceptional point. The critical point in our case arises due to gain competition which is a co-operative effect occurring in a non-equilibrium driven-dissipative system. However, we mention some connections with PT symmetry in our context.

Our treatment follows the analysis of S. Longhi as described in [22]. Near the threshold, before the onset of the gain saturation the evolution of our system following (Eq 2 of the manuscript) can be written as:

$$\mathrm{i}\frac{\partial \Psi}{\partial \xi} = H\Psi \tag{S.39}$$

where, $\Psi = [a_+, {a_-}^*]^T$, represents the vector consisting of the signal and the conjugated idler field. H is given by:

$$H = \begin{bmatrix} -\mathrm{i}\alpha + E_0 & \mathrm{i}W \\ \mathrm{i}W & -\mathrm{i}\alpha - E_0 \end{bmatrix} \tag{S.40}$$

where $W = g$, and $E_0 = -\left[\Delta\phi + \frac{\beta_2}{2}(\delta\omega)^2\right]$. All parameters are described in the main manuscript.

It is to be noted that H exhibits anti-PT symmetry, i.e. $HPT = -PTH$, where P is the parity operator (σ_1, Pauli operator), and T is the time reversal operator. If we redefine the parity operator [22], as $P^{'}$, with $P^{'} = \sigma_3$, then H will also be $P^{'}T$ symmetric, i.e. $P^{'}TH = HP^{'}T$. These symmetries of the Hamiltonian will dictate the eigenvalues in their respective unbroken regimes.

In the unbroken anti-PT symmetry regime, if Ψ is an eigenvector of H with eigenvalue E, then $PT\Psi$ is an eigenvector with eigenvalue $-E^*$. Thus anti-PT symmetry is unbroken when the energy spectrum of H is imaginary.

Similarly, in the unbroken $P^{'}T$ symmetry regime, if Ψ is an eigenvector of H with eigenvalue E, then $P^{'}T\Psi$ is an eigenvector with eigenvalue E^*. Thus $P^{'}T$ symmetry is unbroken when the energy spectrum of H is real.

We can neglect α, by making a non-unitary transformation, and put $\alpha = 0$. Let us consider an uncoupled Hamiltonian (H_0), $H_0 = \Delta\phi - \mathrm{i}\frac{\beta_2}{2}\frac{\partial^2}{\partial t^2}$. Now, we see that $\Phi = \mathrm{e}^{\mathrm{i}\delta\omega t}$ is an eigenvector of H_0, i.e. $H_0\Phi = E_0\Phi$ with eigenvalue E_0, then we can write the eigenvectors ($\Psi_\pm$) and eigenvalues ($E_\pm$) of H as [22]: $\Psi_\pm = [\ \mathrm{i} < \Phi|W\Phi >,\ E_\pm - E_0]^T\ \Phi$, and $E_\pm = \pm\sqrt{E_0^2 - < \Phi|W\Phi >^2}$.

Thus, we see that if $E_0 = 0$, is not an eigenvalue of H_0, i.e. there is no zero-energy modes of H_0, the system is in the unbroken $P^{'}T$ phase.

Therefore, in anomalous dispersion regime, i.e. $\beta_2 < 0$, we can have zero-energy modes for positive detuning values. So, at $\delta\omega = \sqrt{\frac{-2\Delta\phi}{\beta_2}}$, we have $E_0 = 0$, a zero-energy mode, and $P^{'}T$ symmetry can be spontaneously broken. Similarly, one can analyze the normal dispersion regime as well. Thus, at the non-degenerate oscillation frequency $P^{'}T$ symmetry is spontaneously broken.

References

[1] Ryan Hamerly, Alireza Marandi, Marc Jankowski, Martin M Fejer, Yoshihisa Yamamoto, and Hideo Mabuchi. Reduced models and design principles for half-harmonic generation in synchronously pumped optical parametric oscillators. *Physical Review A*, 94(6):063809, 2016.

[2] Kestutis Staliunas and Victor J Sanchez-Morcillo. *Transverse patterns in nonlinear optical resonators*, volume 183. Springer Science & Business Media, 2003.

[3] H Strogatz Steven. Nonlinear dynamics and chaos: with applications to physics, biology, chemistry, and engineering, 1994.

[4] Hermann Haken. Cooperative phenomena in systems far from thermal equilibrium and in nonphysical systems. *Reviews of Modern Physics*, 47(1):67, 1975.

[5] PD Drummond, KJ McNeil, and DF Walls. Non-equilibrium transitions in sub/second harmonic generation. *Optica Acta: International Journal of Optics*, 27(3):321–335, 1980.

[6] Cyril Godey, Irina V Balakireva, Aurélien Coillet, and Yanne K Chembo. Stability analysis of the spatiotemporal lugiato-lefever model for kerr optical frequency combs in the anomalous and normal dispersion regimes. *Physical Review A*, 89(6):063814, 2014.

[7] Michael Cross and Henry Greenside. *Pattern formation and dynamics in nonequilibrium systems.* Cambridge University Press, 2009.

[8] GJ De Valcarcel, Kcstutis Staliunas, Eugenio Roldán, and VJ Sánchez-Morcillo. Transverse patterns in degenerate optical parametric oscillation and degenerate four-wave mixing. *Physical Review A*, 54(2):1609, 1996.

[9] Alireza Marandi, Zhe Wang, Kenta Takata, Robert L Byer, and Yoshihisa Yamamoto. Network of time-multiplexed optical parametric oscillators as a coherent ising machine. *Nature Photonics*, 8(12):937, 2014.

[10] Yutaka Takeda, Shuhei Tamate, Yoshihisa Yamamoto, Hiroki Takesue, Takahiro Inagaki, and Shoko Utsunomiya. Boltzmann sampling for an xy model using a non-degenerate optical parametric oscillator network. *Quantum Science and Technology*, 3(1):014004, 2017.

[11] CD Nabors, ST Yang, T Day, and RL Byer. Coherence properties of a doubly resonant monolithic optical parametric oscillator. *JOSA B*, 7(5):815–820, 1990.

[12] C Fabre, E Giacobino, A Heidmann, and S Reynaud. Noise characteristics of a non-degenerate optical parametric oscillator-application to quantum noise reduction. *Journal de Physique*, 50(10):1209–1225, 1989.

[13] AS Villar, Mi Martinelli, and P Nussenzveig. Testing the entanglement of intense beams produced by a non-degenerate optical parametric oscillator. *Optics communications*, 242(4-6):551–563, 2004.

[14] W. Zeller E. Tirapegui. *Instabilities and Nonequilibrium Structures V.* Springer, 1996. http://dx.doi.org/10.1007/978-94-009-0239-8.

[15] A Gatti and L Lugiato. Quantum images and critical fluctuations in the optical parametric oscillator below threshold. *Physical Review A*, 52(2):1675, 1995.

[16] R Medeiros de Araújo, Jonathan Roslund, Yin Cai, Giulia Ferrini, Claude Fabre, and Nicolas Treps. Full characterization of a highly multimode entangled state embedded in an optical frequency comb using pulse shaping. *Physical Review A*, 89(5):053828, 2014.

[17] Colin J McKinstrie, Stojan Radic, and Andrew R Chraplyvy. Parametric amplifiers driven by two pump waves. *IEEE Journal of Selected Topics in Quantum Electronics*, 8(3):538–547, 2002.

[18] Andrea Armaroli and Stefano Trillo. Collective modulation instability of multiple four-wave mixing. *Optics letters*, 36(11):1999–2001, 2011.

[19] Julien Fatome, Christophe Finot, Andrea Armaroli, and Stefano Trillo. Observation of modulationally unstable multi-wave mixing. *Optics Letters*, 38(2):181–183, 2013.

[20] Joshua E Rothenberg. Modulational instability of copropagating frequencies for normal dispersion. *Physical review letters*, 64(7):813, 1990.

[21] Peter D Drummond and Kaled Dechoum. Universality of quantum critical dynamics in a planar optical parametric oscillator. *Physical review letters*, 95(8):083601, 2005.

[22] Stefano Longhi. Pt symmetry and antisymmetry by anti-hermitian wave coupling and nonlinear optical interactions. *Optics letters*, 43(16):4025–4028, 2018.

[23] Xiang Guo, Chang-Ling Zou, Hojoong Jung, and Hong X Tang. On-chip strong coupling and efficient frequency conversion between telecom and visible optical modes. *Physical review letters*, 117(12):123902, 2016.

[24] Yanne K Chembo. Quantum dynamics of kerr optical frequency combs below and above threshold: Spontaneous four-wave mixing, entanglement, and squeezed states of light. *Physical Review A*, 93(3):033820, 2016.

[25] Andrey B Matsko, Anatoliy A Savchenkov, Dmitry Strekalov, Vladimir S Ilchenko, and Lute Maleki. Optical hyperparametric oscillations in a whispering-gallery-mode resonator: Threshold and phase diffusion. *Physical Review A*, 71(3):033804, 2005.

[26] German J De Valcarcel, Giuseppe Patera, Nicolas Treps, and Claude Fabre. Multimode squeezing of frequency combs. *Physical Review A*, 74(6):061801, 2006.

[27] L Kramer, HR Schober, and W Zimmermann. Pattern competition and the decay of unstable patterns in quasi-one-dimensional systems. *Physica D: Nonlinear Phenomena*, 31(2):212–226, 1988.
